# Advancements in bottom-up synthesis routes for monolayer graphene: a review

**DOI:** 10.1098/rsos.260473

**Published:** 2026-10-21

**Authors:** Muhammad A. Awan, Szymon Abrahamczyk, Maria Hasan, Pavel Kuzník, Maria Zdończyk, Alicja Bachmatiuk, Mark H. Rümmeli

**Affiliations:** ^1^ Centre for Nanotechnology, Faculty of Materials Sciences and Technology, Vysoka skola banska-Technicka univerzita Ostrava, Ostrava, Moravian-Silesian Region, Czech Republic; ^2^ Electron Beam Emergent Additive Manufacturing (EBEAM) Centre, Centre for Nanotechnology (CNT), Centre for Energy and Environmental Technologies (CEET), Vysoka skola banska-Technicka univerzita Ostrava, Ostrava, Moravian-Silesian Region, Czech Republic; ^3^ Centre for Nanotechnology (CNT), Centre for Energy and Environmental Technologies (CEET), Vysoka skola banska-Technicka univerzita Ostrava, Ostrava, Moravian-Silesian Region, Czech Republic; ^4^ Faculty of Materials Science and Technology, Vysoka skola banska-Technicka univerzita Ostrava, Ostrava, Moravian-Silesian Region, Czech Republic; ^5^ Faculty of Chemistry, Wrocław University of Science and Technology, Wrocław, Poland; ^6^ Institute for Materials Chemistry, Leibniz Institute for Solid State and Materials Research Dresden, Dresden, SN, Germany; ^7^ Soochow Institute for Energy and Materials Innovation, Soochow University, Suzhou, People's Republic of China; ^8^ School of Smart Materials and Future Energy, Anhui Normal University, Wuhu 214002, People's Republic of China

**Keywords:** monolayer graphene, bottom-up synthesis, chemical vapour deposition, epitaxial growth on SiC, arc discharge, molecular assembly, single-atom catalysts, machine learning

## Abstract

The synthesis of monolayer graphene has progressed from exfoliation to bottom-up strategies, enabling increasing atomic-level control. This review compares key routes, chemical vapour deposition (CVD), SiC epitaxy, arc discharge, molecular assembly and single-atom catalysts (SACs) and evaluates their mechanisms, scalability and quality bottlenecks. CVD remains the most mature and scalable approach, delivering centimetre-scale-single-crystal films through optimized substrates, roll-to-roll annealing and dry transfer, yet transfer-induced defects and wafer-scale non-uniformity persist. SiC epitaxy enables transfer-free, wafer-scale graphene with well-aligned domains, but requires high temperatures and costly wafers. Arc discharge offers catalyst-free production of crystalline graphene flakes, although monolayer yield and purity remain limited. Molecular assembly provides unmatched structural programmability and atomic precision in nanoribbons and nanographene islands, typically restricted to micrometre scales and stringent conditions. SAC-mediated growth represents a precision limit, where isolated metal atoms catalyse single-carbon insertion or removal at graphene edges, though current demonstrations remain largely single-event observations. Complementing these routes, artificial intelligence/machine learning is increasingly used for parameter optimization, predictive modelling of domain size/uniformity/defects and automated quality control (convolutional neural network/ generative modelling and YOLO-based defect detection). Overall, industrially viable, defect-free monolayer graphene will require combining scalable growth, transfer-free integration, atomic-precision and AI-enabled closed-loop optimization.

## 1. Introduction

As atomically thin sheets of *sp²*-bonded carbon, graphene is a paradigm two-dimensional material combining exceptional electronic, mechanical and chemical properties. Its linear band dispersion near the Dirac points produces extraordinarily high carrier mobilities, enabling transport phenomena that are valuable from both scientific and technological perspectives [1,2]. Single graphene sheets exhibit an ultra-high Young's modulus as well as remarkable tensile strength and flexibility; their large surface area and chemically tuneable basal plane are attractive features for sensing, catalysis and development of composite reinforcement [3,4]. These attributes have translated into a broad range of functional platforms: the high specific surface area and chemically active basal plane of graphene make three-dimensional graphene architectures effective supports for heterogeneous electrocatalysis [5], while its mechanical compliance and electronic sensitivity underpin flexible pressure- and strain-sensing devices [6]. The growing library of graphene derivatives, together with their defect landscapes, further broadens this property-application space [7]. These unique advantages, however, are strongly contingent on preserving an uninterrupted monolayer lattice that contains sparse or no defects. Even subtle variations in thickness, stacking order or grain boundary density can change the electronic band structure and mechanical response, possibly reducing device effectiveness [8–10].

The distinction between monolayer and multi-layer graphene is more than nominal. A single atomic layer retains the Dirac cone and ambipolar electronic response that underpin many proposed device concepts. By contrast, bilayer and few-layer graphene (FLG) exhibit markedly altered band dispersions, tuneable gaps under external fields and reduced electron mobility, thereby compromising high-frequency and quantum device performance [11,12]. Currently, the main challenge in graphene synthesis is not producing the carbon sheets but rather delivering large-area, continuous monolayers with controlled domain orientation, minimal grain boundaries and reproducible surface chemistry [13,14]. Achieving such films requires the precise control of nucleation, lateral domain growth, defect formation and post-growth processing. These requirements, in turn, expose the limitations of many existing production routes and motivate renewed emphasis on growth mechanisms and post-growth processing [15,16].

Historically, graphene has been synthesized using two methods: top-down exfoliation of bulk graphite and bottom-up assembly from molecular or atomic precursors [17]. Top-down techniques, such as mechanical exfoliation, yield flakes of near-ideal crystallinity that are indispensable for fundamental studies; however, their stochastic nature and limited lateral extent make them impractical for scalable device fabrication [18,19]. Chemical routes that oxidize and then reduce graphite can generate macroscopic films and dispersions, yet they also introduce irreversible structural damage and oxygen functional groups that degrade the electrical and mechanical performance [20]. Liquid-phase and electrochemical exfoliation methods have higher throughput, but they suffer from wide thickness distributions and a persistent trade-off between yield and structural integrity [21,22]. These shortcomings underscore the necessity of bottom-up strategies that assemble graphene directly onto substrates under engineered conditions favouring single-layer formation and controllable domain evolution [23].

The main bottom-up synthetic methods include catalytic chemical vapour deposition (CVD) [18], epitaxial growth on silicon carbide (SiC) [24], plasma/arc discharge [25] and on-surface molecular assembly [26]. Each method offers distinct pathways for monolayer formation and therefore different opportunities for process control. CVD on metals with low carbon solubility (e.g. Cu) usually exploits surface-limited growth; it can produce continuous monolayers on a scale of centimetres when the nucleation density and carbon flux are judiciously managed. However, this method is sensitive to substrate crystallography, contamination and transfer-related degradation [27]. The epitaxial decomposition of SiC affords direct growth on insulating wafers and tends to produce high-quality monolayers in localized regimes; however, extreme temperatures and careful buffer-layer engineering are required to suppress multi-layer nucleation [28]. Arc- and plasma-based vaporization routes generate abundant carbon fragments that can reassemble into thin graphitic sheets. Nevertheless, controlling the number of layers and film uniformity is challenging without the precise regulation of cooling kinetics and gas chemistry [25]. Molecular assembly and on-surface synthesis deliver atomic precision, enabling designer edge structures and nanoporous networks through tailored precursors and cyclodehydrogenation reactions, even though they are presently limited in the lateral scale and throughput [26,29]. Thus, each route supplies unique levers for monolayer control, and a critical comparison of their underlying kinetics, substrate interactions and scalability is central to advancing wafer-scale monolayer production.

A particularly promising and emerging direction is single-atom catalyst (SAC)-mediated growth, in which isolated transition metal atoms anchored to supports act as discrete catalytic centres for carbon incorporation. Recent atomically resolved experiments and modelling indicate that SACs can modulate local nucleation density and edge chemistry at the single-atom level, enabling stepwise carbon addition with potentially lower activation barriers and fewer parasitic multi-layer nucleation events than conventional nanoparticle catalysts [30–32]. While the SAC strategies are still at an early stage, their capacities to decouple nucleation density from macroscopic catalyst morphology and to tailor the balance between growth and etching through the choice of metal and coordination environment position them as a distinctive catalyst engineering paradigm for achieving uniform monolayers [30]. Integrating SACs with established bottom-up platforms (e.g. embedding isolated atoms on Cu foils or within nitrogen-doped carbon supports for CVD) is an attractive pathway toward combining atomic precision with industrial production.

The controlled synthesis of high-quality monolayer graphene is the cornerstone of two-dimensional materials research, yet existing reviews tend to examine only specific methodologies, particularly CVD, which has been surveyed extensively. For example, Liu *et al.* [33] explore ‘precise synthesis’ targets (single-crystal graphene, AB-stacked bilayer, etched and clean graphene) but remain within CVD, while Huang *et al.* [34] focus on technological progress and challenges in CVD, such as flatness and layer number control. Bahri & Ma [35] provide broad CVD surveys encompassing low-pressure and atmospheric-pressure variants and their applications, and Muñoz-Vargas *et al.* [36], explicitly connect CVD production with AI-enabled quality analysis. By contrast, the present review deliberately adopts a monolayer-centred, routes-comparative perspective, it positions CVD alongside SiC epitaxy, arc discharge plasma synthesis, on-surface molecular assembly and highly emergent SAC strategies within a unified mechanism to process structure towards defect/quality to scalability framework. This alignment enables direct comparison of how nucleation control, carbon feedstock delivery, substrate/edge energetics and defect formation determine monolayer fidelity and manufacturability across all methods. Moreover, building on this cross-platform comparison, by treating artificial intelligence/machine learning (AI/ML) as a cross-cutting layer that supports parameter optimization, predictive modelling of film properties (domain size, uniformity and defect density) and automated defect/damage assessment, our review proposes a practical roadmap toward reproducible, large-area, high-quality monolayer graphene, thereby extending recent AI-driven approaches and integrating them across all bottom-up synthesis routes.

To address these challenges, we compare recent advances in bottom-up monolayer graphene growth, emphasizing mechanistic understanding, process–structure relationships and emerging catalytic strat egies and routes that offer enhanced thickness control and uniformity over a large area, including machine learning (ML) and artificial intelligence (AI). By critically evaluating each synthesis route's ability to control nucleation, domain coalescence and defect formation, we highlight cross-cutting solutions, ranging from substrate crystallography and surface pretreatment to *in situ* diagnostics and SAC engineering that bridge laboratory demonstrations and scalable manufacturing. Figure 1 schematically depicts the synthesis routes discussed here. By placing conventional and novel bottom-up approaches next to each other, this review aims to provide a practical roadmap for achieving reproducible, high-quality monolayer graphene suitable for next-generation devices.

**Figure 1.**
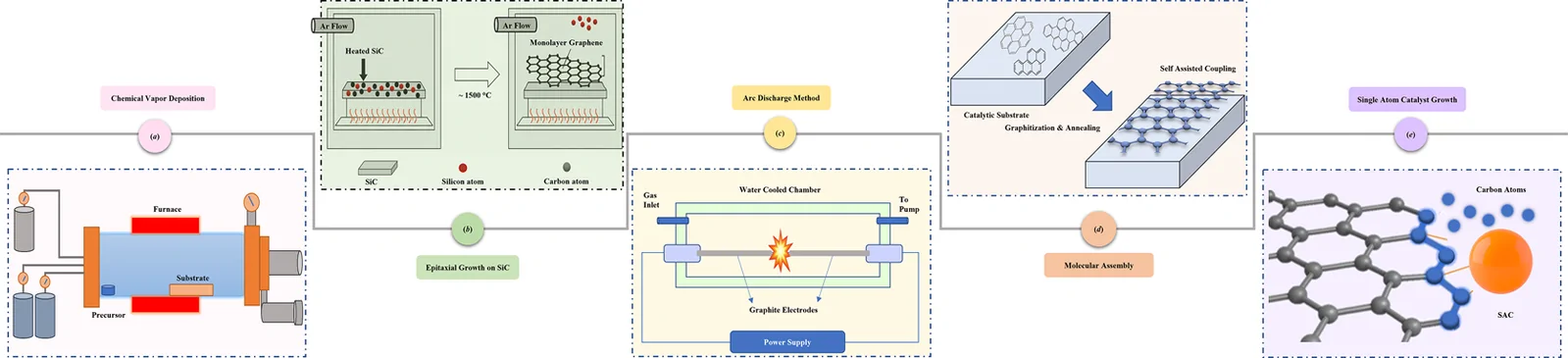
Illustrations of different bottom-up synthesis strategies to produce monolayer graphene from carbon precursors.

## 2. Bottom-up routes for monolayer graphene synthesis

### 2.1. Chemical vapour deposition method

CVD remains the most advanced and industrially relevant bottom-up method for synthesizing large-area, continuous monolayer graphene [37]. By contrast to exfoliation-based routes that mechanically remove sheets from bulk graphite (pioneered by Novoselov *et al.* [38]), Li *et al.* [39] used low-pressure CVD (LP-CVD) to reliably and reproducibly synthesize large-area, high-quality single-layer graphene (SLG) films on the Cu substrate. This represents a major breakthrough in scalable graphene production.

CVD builds graphene atom by atom on catalytic substrates, thereby offering precise control over nucleation, growth kinetics and layer number. For electronic and sensor applications, CVD enables the creation of monolayer graphene with a large surface area and consistent crystal structure [40]. Over the past decade, it has become the dominant technique for targeting monolayer graphene owing to the unique integration of (i) scalable designs [41], (ii) tuneable properties [42], and (iii) substrate engineering strategies capable of suppressing multi-layer formation [43]. Despite its maturity, however, achieving reproducible uniform monolayers across wafer-scale areas remains challenging and depends critically on the interplay among substrate crystallography, carbon precursor decomposition and growth thermodynamics. Understanding these interactions is key to developing CVD methods for reliable monolayer production.

#### 2.1.1. Engineering of Cu(111) and alternative substrates for wafer-scale monolayer graphene growth

The substrate plays a pivotal role in determining whether CVD yields a uniform monolayer graphene film. A schematic of the CVD set-up is shown in figure 2*a*. Key substrate factors, including the crystallographic structure, impurity levels, surface roughness and carbon solubility, directly influence nucleation and layer uniformity during graphene growth [48]. A wide range of metal and non-metal substrates have been explored to enhance monolayer selectivity [49,50]. Commercial polycrystalline Cu foils are the most common substrate owing to their low cost and, more importantly, low carbon solubility that favours monolayer graphene. However, their heterogeneous surfaces with grain boundaries, high roughness and residual surface contamination require careful pretreatment to suppress multi-layer patches and orientational disorder between graphene domains [51,52]. Single-crystal Cu, particularly the (111) surface, has emerged as an ideal substrate owing to its hexagonal lattice symmetry closely matching that of graphene. The low carbon solubility in Cu and the fully filled three-dimensional shell enable weak graphene–substrate interactions, stabilizing monolayer growth while minimizing re-nucleation. As a result, uniform graphene monolayers can be grown over more than 90% of the surface area [44]. Once the Cu(111) surface is passivated by a graphene layer, the absence of catalytic sites suppresses further nucleation. More broadly, recent surveys of single-crystalline two-dimensional materials and (111)-oriented metal foils (Cu, Ni, Co, Pt and Pd) underscore that the crystallographic perfection of the catalyst foil is decisive for large-area, defect-controlled monolayer growth [53]. However, sporadic secondary-layer flakes may still appear, often originating from organic surface contaminants, such as residual grease or anti-corrosive agents applied during foil manufacturing [54]. Researchers have successfully removed surface carbon atoms and suppressed secondary flake formation by thermal annealing at temperatures near the melting point of Cu (approx. 1060°C) in an H₂-rich environment, enabling cleaner monolayer coverage [55–57].

**Figure 2.**
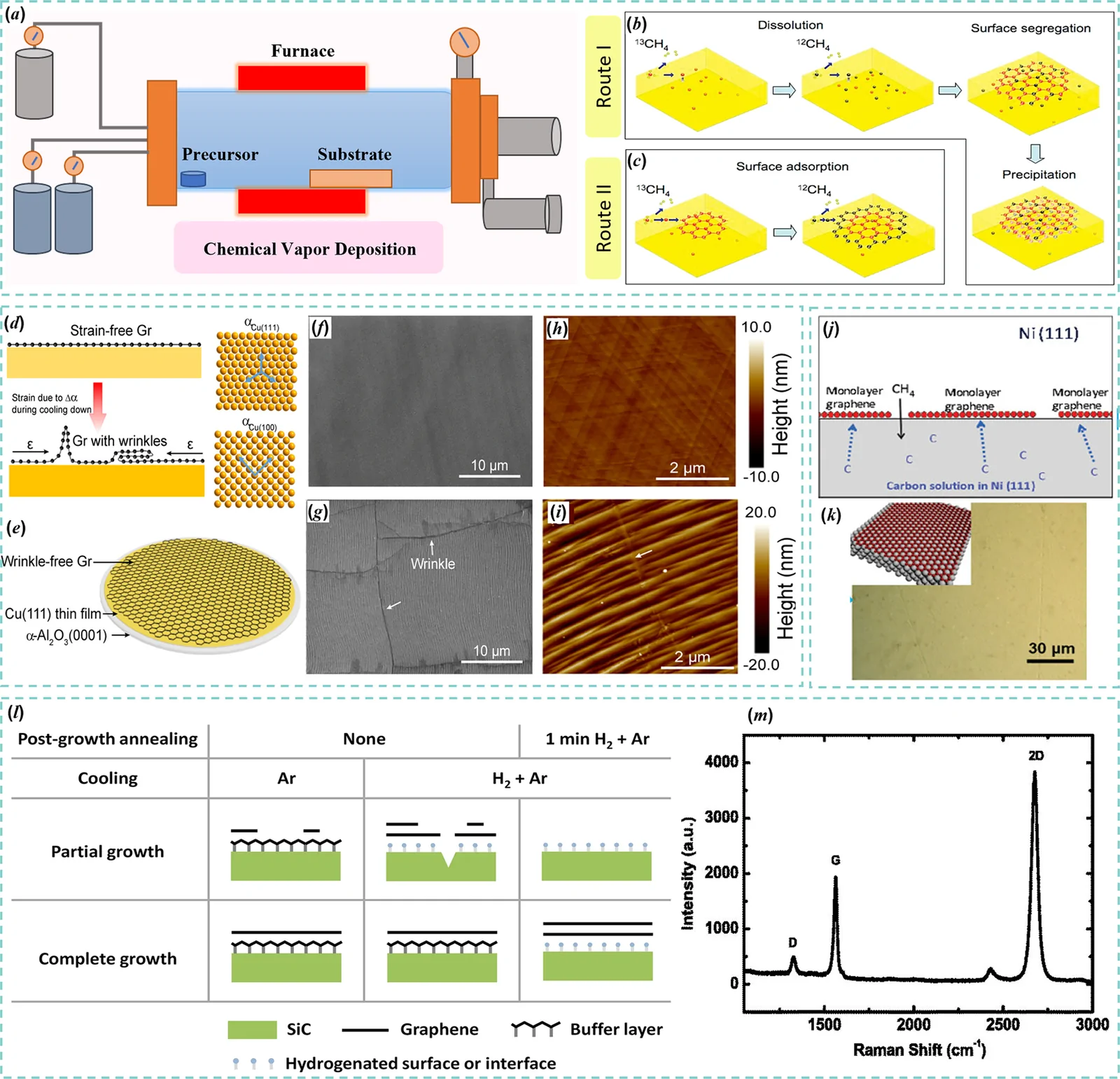
(*a*) Schematic of the typical CVD set-up. (*b*,*c*) Graphene with (*b*) randomly mixed isotopes, possibly because of surface segregation and/or precipitation and (*c*) separated isotopes, possibly by surface adsorption. Reproduced with permission [44]. Copyright 2009, Am. Chem. Soc. (*d*) Schematic of the wrinkle formation process of graphene (Gr) grown on a substrate. The black arrow shows compressive strain induced by thermal mismatch during cooling with the crystal face models of Cu(111) and Cu(100). (*e*) Schematic of wrinkle-free graphene grown on Cu(111) thin film epitaxial on sapphire. (*f*,*g*) scanning electron microscopy (SEM) images and (*h*,*i*) atomic force microscopy (AFM) images of graphene grown on the Cu(111) thin film and Cu(100) foil, respectively. Reproduced with permission [45]. Copyright 2017, Am. Chem. Soc. (*j*) Schematic of the graphene growth mechanism on Ni(111). (*k*) Optical image of graphene grown on Ni(111) after the CVD process. Inset: three-dimensional diagrams of the graphene layer on Ni(111), showing single-layer growth. Reproduced with permission [46]. Copyright 2017, Am. Chem. Soc. (*l*) Structures of partial and complete layers after different cooling protocols. (*m*) Raman spectrum of monolayer graphene grown on SiO_2_ upon 532 nm laser excitation. Reproduced with permission [47]. Copyright 2017, Nature.

Graphene domains are influenced by the topography and facets of the Cu substrate [58,59]. Recent breakthroughs have enabled large-area single-crystal Cu(111) foils and films. For instance, roll-to-roll annealing methods converted polycrystalline Cu tapes to nearly single-crystalline Cu(111) with a length of over 1 m and CVD growth on these films yielded continuous monolayer graphene with an extraordinary domain size [60]. As illustrated in figure 2*d*–*i*, wrinkle formation in graphene grown on Cu is primarily driven by compressive strain that develops during cooling owing to thermal expansion mismatch. Comparative studies revealed that graphene grown on single-crystal Cu(111) exhibited a uniform, wrinkle-free morphology owing to compact lattice and minimal anisotropic contraction (figure 2*e*), whereas that grown on Cu(100) foils contained abundant wrinkles and surface roughness, as confirmed by the scanning electron microscopy (SEM) images (figure 2*f*,*g*) and atomic force microscopy (AFM) images (figure 2*h*,*i*). This observation demonstrates the superiority of Cu(111) thin films for achieving ultraflat, high-quality monolayer graphene suitable for device-grade applications [45]. Similarly, Cu films deposited on sapphire and annealed adopt a continuous (111) texture. Graphene grown on Cu(111)/sapphire possessed centimetre-scale single-crystal domains and excellent monolayer uniformity. Overall, engineered Cu(111) substrates virtually remove grain boundaries from both the metal and graphene, producing monolayer graphene having not only a uniform layer number but also identical crystallographic orientation across wafer-scale areas [61].

Compared with Cu, metal substrates that efficiently catalyse hydrocarbon decomposition suffer from high carbon solubility, which fundamentally complicates monolayer control. For example, carbon readily dissolves at CVD temperatures in bulk Ni and later segregates during cooling, leading to the precipitation of multiple graphene layers. Consequently, early attempts at CVD on Ni substrates typically produced two-to-three layer graphene films with poor uniformity and crystallinity. Isotopic labelling experiments with sequential dosing of ¹²CH₄ and ¹³CH₄ provided mechanistic insights into Ni: bulk carbon dissolution followed by surface segregation yields isotopically mixed multi-layers (figure 2*b*), underscoring the inherent limitations of Ni for selective monolayer growth [62]. Nevertheless, the use of single-crystal Ni(111) has been shown to significantly improve the monolayer/bilayer coverage in relation to that of polycrystalline Ni (91.4 versus 72.8%, figure 2*j*,*k*), because the grain boundaries in polycrystalline Ni act as multi-layer nucleation sites [46].

Non-metallic substrates, such as SiC, offer alternative routes to monolayer graphene. However, post-growth cooling must be carefully controlled. Figure 2*l* shows that varying the amounts of hydrogen and argon during cooling can lead to either beneficial hydrogen intercalation or unwanted graphene etching. Pure argon cooling suppresses the effects of hydrogen and preserves the monolayer, whereas hydrogen-rich cooling can hydrogenate the buffer layer or etch incomplete layers. These effects are especially pronounced when graphene coverage is partial, underscoring the importance of post-growth atmosphere in preserving monolayer integrity on non-metallic substrates [47]. The characteristic of the Raman spectrum in figure 2*m* confirms the monolayer nature of graphene synthesized under these optimized conditions [63].

#### 2.1.2. Alloy substrates as tailored catalysts for uniform single-layer graphene synthesis

Alloy substrates have emerged as a powerful class of engineered catalysts in CVD graphene synthesis because their tuneable carbon solubility and surface chemistry enable the precise modulation of growth kinetics, domain structure and monolayer selectivity [52]. Notably, even a small amount of added Ni can drastically accelerate growth owing to a lower energy barrier for methane decomposition: single-crystal Cu/Ni(111) foils with approximately 1–9% Ni achieved full monolayer coverage in 5 minutes, whereas pure Cu(111) required approximately 60 minutes [64]. These Cu-Ni alloy substrates also promoted large, highly oriented domains. Indeed, near-single-crystal monolayer films were obtained on Cu/Ni(111) with an optimized Ni content [52]. In Ni-Mo binary catalysts, Mo acts as a carbon ‘sink’ that forms stable carbides, suppressing the usual carbon precipitation on Ni and enabling strict SLG growth, achieving 100% surface coverage across a wide process window. Here, Ni provides active sites for hydrocarbon cracking, whereas Mo sequesters dissolved carbon and thus prevents multi-layer formation [65]. In another alloy approach, Au-Ni thin films significantly lowered the growth temperature, enabling plasma-enhanced CVD (PECVD) to yield monolayer graphene at approximately 450°C. This is because Au in the alloy dilutes the carbon uptake, while Ni maintains the catalytic activity [66]. Experiments on Cu-Pt alloy foils further demonstrated how alloy composition can be leveraged to tune the number of layers and crystallinity of graphene. Raman analysis of graphene grown on Cu/Pt foils with varying weight ratios revealed that both monolayer and bilayer graphene could be selectively obtained. At low CH₄ flow rates (approx. 1.5 standard cubic centimetres per minute (sccm)) and reduced growth temperatures (as low as 750°C), the alloy exhibited enhanced catalytic activity and allowed time-controlled growth, highlighting its promise for low-temperature, layer-engineered synthesis [67]. In the aforementioned alloy designs, tailoring the metal–metal combinations allows researchers to balance carbon solubility and catalytic activity in CVD processes, thereby accelerating growth, lowering processing temperatures and achieving uniform monolayer graphene.

#### 2.1.3. Emerging strategies for transfer-free graphene synthesis on insulating and metal-assisted substrates

Conventional CVD graphene synthesis relies on metal catalysts (e.g. Cu and Ni) to crack carbon precursors and template graphene growth. This necessitates a transfer step to move graphene onto insulating device substrates, and this step often introduces cracks, residues and defects [68]. Comprehensive reviews of transfer-free and direct growth on rigid and flexible substrates [69] and of strategies for moving two-dimensional films from imperfect to near-perfect transfer [70] detail how these defects can be mitigated. Transfer can be bypassed by directly growing monolayer graphene on the target insulating substrate; however, this has historically yielded polycrystalline films with high defect density, low carrier mobility and high sheet resistance [71]. Although direct, catalyst-free graphene growth on insulating and semiconducting substrates is possible, the inherently low catalytic activity of non-metal surfaces leads to low growth rates. By contrast, various transition metals are known to efficiently catalyse precursor decomposition and significantly accelerate graphene formation during CVD [72,73]. To bridge the gap between catalytic performance and substrate compatibility, metal-assisted graphene growth has emerged as a promising alternative. Levendorf *et al.* [74] first demonstrated this approach by using an evaporated Cu film as a sacrificial catalyst to decompose the carbon precursor, enabling large-area SLG growth directly on a metal-free substrate and fabricating uniform transistor arrays with uniform electrical properties. Similarly, Han *et al.* [61] grew an ultraflat single-crystal monolayer across a sapphire wafer by annealing a Cu film *in situ* on a Cu(111) catalyst and growing graphene at the Cu/sapphire interface. Afterwards, Cu was thermally peeled off via liquid-nitrogen quenching, leaving behind a single-crystal graphene sheet on the insulating substrate. Dong *et al.* [75] presented a transfer-free method for directly synthesizing monolayer graphene on insulating substrates using a sacrificial Ni catalyst film, as schematically illustrated in figure 3*IA*. After CVD growth and coating a polymer support, Ni was etched while the graphene remained pinned to the underlying SiO₂ surface, thus avoiding manual transfer and photoresist contamination (figure 3*IB*–*ID*). Raman spectra confirm the monolayer quality and uniformity of graphene on various substrates (figure 3*IE*,*IF*), while patterned growth without lithographic damage is indicated in figure 3*IG*. Wang *et al.* [76] demonstrated a Ge-assisted transfer-free CVD method for directly synthesizing patterned monolayer graphene on dielectric substrates (figure 3*II*). The time-dependent evolution of Ge sublimation (figure 3*IIA1*–*E1*) and Raman mapping of Ge peak intensity at different growth times (figure 3*IIA2*–*E2*) reveal that the optimal CVD duration enables complete Ge removal while yielding high-quality monolayer graphene, as confirmed by Raman mapping of the I_2D_/I_G_ ratio. The Raman spectra (figure 3*IIF*,*IIG*) further confirm the emergence of defect-free graphene and the disappearance of the Ge signal with increasing growth time. Zhang *et al.* [77] introduced a rapid, transfer-free CVD method for the direct synthesis of monolayer graphene on ultrathin free-standing Pd leaves, leveraging the latter's exceptional thermal stability and low grain boundary density (figure 3*IIIA*). High-quality graphene was grown within 1 minute owing to the enhanced grain growth kinetics on Pd, as confirmed by Raman spectra with a sharp 2D peak and a low I_D_/I_G_ ratio (mean value: 0.076), indicating minimal defects (figure 3*IIIB*,*C*). The resulting graphene/Pd hybrid exhibited significantly enhanced mechanical properties with a 59% increase in surface modulus, making it promising for advanced coating applications. Hagendoorn *et al.* [78] demonstrated a transfer-free CVD method for directly growing monolayer graphene on SiO/Si substrates by using a patterned Pt thin film as a catalytic seed layer. The Pt film was annealed at 1000°C for 60 minutes either under atmospheric conditions or in nitrogen gas at 1 bar, resulting in a typical Pt grain size of approximately 0.5–1.0 µm according to the SEM images (figure 3*IVA*,*B*) and AFM images (figure 3*IVC*,*D*). Graphene growth occurred at the buried interface between Pt and SiO₂, enabling the coverage of a full 4 inch wafer after Pt removal (figure 3*IVE*), as confirmed by the Raman spectra in figure 3*IVF*. Moreover, Ta blocking structures were implemented to spatially control graphene nucleation, offering a lithography-compatible route for site-selective, high-quality graphene synthesis directly on insulating substrates.

**Figure 3.**
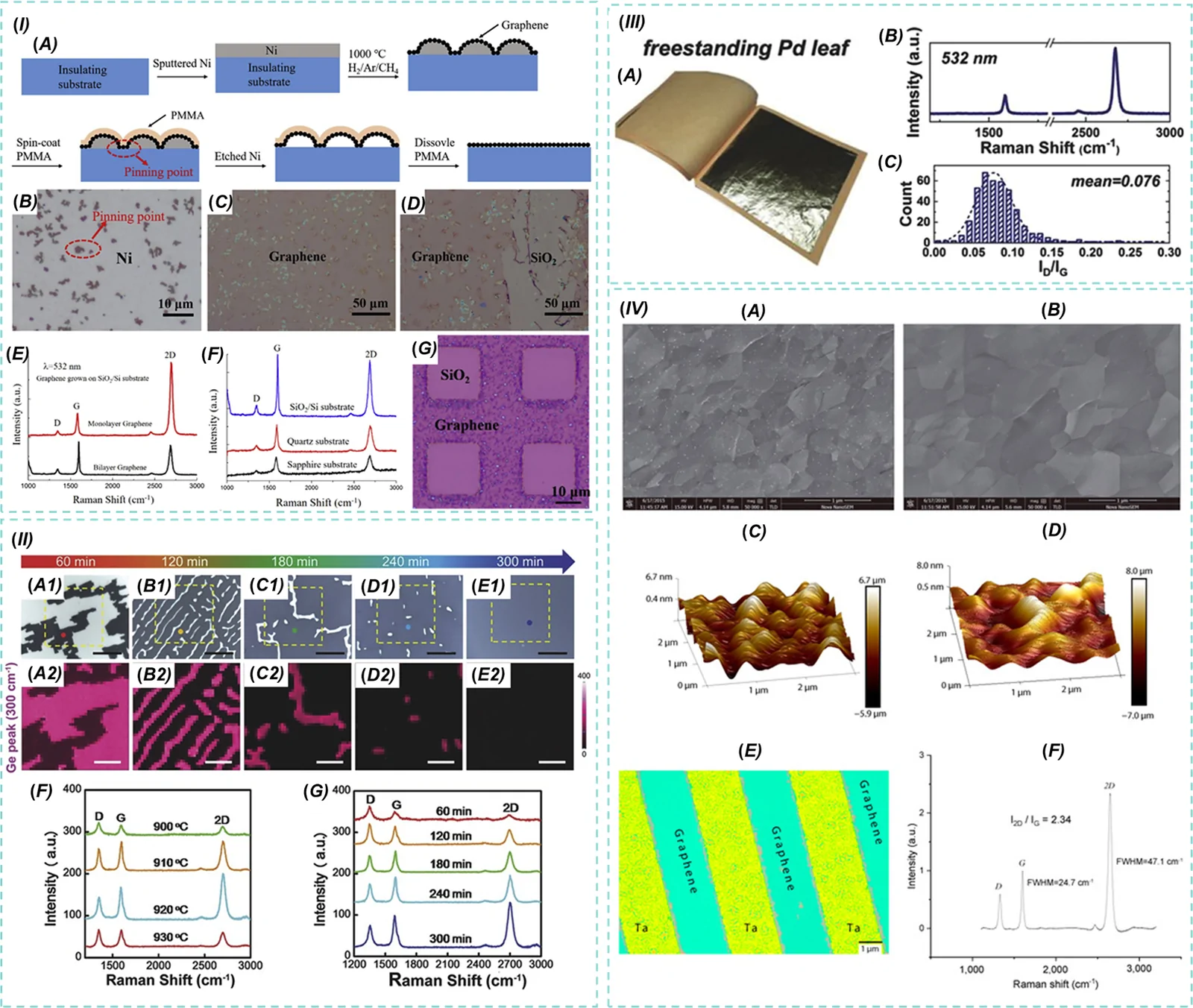
(*I*) Direct growth of transfer-free and lithography-free graphene on insulating substrates, using Ni as a sacrificial metal catalyst. (*A*) Schematic of the direct growth of transfer-free graphene on insulating substrates by the subsequent etching of the sacrificial metal catalyst. (*B*–*D*) Optical images of (*B*) Ni morphology after graphene growth and (*C* and *D*) graphene after Ni catalyst etching. (*E*) Typical Raman spectra of graphene grown on SiO_2_/Si substrates. (*F*) Raman spectra of graphene grown on three different substrates (SiO_2_/Si, quartz and sapphire). (*G*) Optical image of the patterned sample after graphene growth. Reproduced with permission [75]. Copyright 2018, IOP Publishing Ltd. (*II*) High-quality graphene grown on thin Ge films. (*A*–*E*) Evolution of Ge vaporization and graphene formation during CVD. (*A1*–*E1*) Optical micrographs of graphene grown on SiO_2_/Si at 920°C for different times. Scale bar: 20 µm. (*A2*–*E2*) Raman maps of Ge peak intensity as a function of growth time. The mapped areas are denoted in (*A1*)–(*E1*) by dashed yellow squares. Scale bar: 10 µm. (*F*) Raman spectra of graphene grown at different temperatures for 300 minutes. (*G*) Raman spectra of graphene grown by CVD at 920°C for different times. Reproduced with permission [76]. Copyright 2017, Wiley-VCH. (*III*) Graphene growth on Pd leaf substrate. (*A*) Photograph of a Pd leaf (140 mm × 140 mm × 150 nm, which is commercially available as free-standing sheets laminated between low surface-energy paper. (*B*) Raman spectrum (532 nm laser) of the as-grown graphene sheet after separation from Pd leaf and transfer to a SiO_2_/Si substrate. (*C*) Histogram of I_D_/I_G_ ratios obtained from Raman maps (23 × 23 µm). Reproduced with permission [77]. Copyright 2018, Wiley-VCH. (*IV*) SEM and AFM images of the Pt seed layer after annealing. (*A*,*B*) SEM images of (*A*) an area with a grain size of approximately 0.5 µm after annealing in nitrogen gas and (*B*) an area with a grain size of approximately 1.0 µm after annealing under atmospheric conditions. (*C*,*D*) Respective surface roughness of (*A*) and (*B*) determined by AFM over an area of 3 × 3 µm. (*E*) Optical micrograph of a wafer with patterned Ta blocking structures (yellow) and transfer-free CVD graphene (turquoise). (*F*) Raman spectrum of CVD graphene layer between Ta blocking structures. Reproduced with permission [78]. Copyright 2022, MDPI.

Compared with direct growth on metal-free substrates, metal-assisted CVD offers the dual advantages of metal-catalysed hydrocarbon decomposition and direct graphene formation on the target substrate. This section highlights recent progress in transfer-free growth of monolayer graphene on insulating substrates assisted by metals. By contrast, in the absence of catalysts, the direct growth of graphene on insulating substrates relies primarily on external thermal resources to overcome the energy required for feedstock decomposition [50]. Fanton *et al.* [79] demonstrated the feasibility of growing graphene at elevated temperatures on sapphire substrates. More recent research has improved the film quality by tailoring the substrate properties and growth conditions. For example, Chen *et al.* [80] achieved wafer-scale monolayer graphene on sapphire by using electromagnetic induction heating to reach approximately 1400°C, greatly enhancing methane pyrolysis and surface carbon diffusion. This new growth strategy enabled the large-scale production of high-quality graphene films with an expanded para meter space. A graphite sample holder accommodating the sapphire substrates was placed in the reaction chamber and heated using a surrounding induction coil [80]. Dong *et al.* [75] employed the same process to grow graphene directly on SiO₂ and found that graphene adhered to the substrate with minimal mechanical handling, thereby avoiding cracks or tears. Because the Ni catalyst was present during growth, as-grown graphene exhibited higher quality in terms of defect density and grain size than graphene grown on metal-free SiO₂. The Ni thickness and growth temperature can be tuned to favour monolayer formation—for example, excessively thick Ni or high C solubility can induce multiple layers.

#### 2.1.4. From surface decontamination to self-alignment: mechanistic pathways enabling wafer-scale single-crystal graphene on insulating substrates

Achieving wafer-scale single-crystal graphene on insulators requires careful control of nucleation and domain orientation through several complementary mechanisms. Surface decontamination and preparation are crucial steps. For example, the hydrogen pretreatment of sapphire yields atomically clean stepped surfaces that reduce wrinkling and defect density during graphene growth [81]. Similarly, the mild oxidation or plasma treatment of Cu catalysts can passivate the active sites, remove impurities, suppress random nucleation and enable the formation of millimetre-scale single-crystal domains [82]. Step-edge-guided growth on vicinal or prepatterned surfaces can further enforce alignment. Notably, graphene grown on a 15° miscut Ge(001) wafer, which contains a dense array of step edges, exhibits unidirectional island alignment owing to suppressed nucleation on specific terrace facets [83]. Ge-assisted growth on a dielectric (Ge on a Si wafer) highlights how a semiconductor substrate with catalytic activity can yield single-oriented graphene without requiring a metal foil. More generally, thin catalytic films of Ge can be deposited on amorphous insulators, such as SiO₂ and then partly evaporated during CVD, acting as a transient catalyst and directly leaving graphene on the dielectric substrate [66]. Controlled layer-by-layer thinning [84] and patterned (‘art’) etching [85] of graphene offer complementary top-down handles for tailoring layer number and geometry.

Zhang *et al.* [86] used a CO₂-assisted CVD strategy to remove intrinsic amorphous carbon contamination from graphene surfaces (figure 4*a*). During standard CVD growth, amorphous carbon accumulates on graphene, as confirmed by transmission electron microscopy (TEM) (figure 4*b*) and a broadened C–C bond angle distribution I (figure 4*c*). Mild CO₂ treatment selectively oxidizes and etches this disordered carbon without damaging the underlying lattice, yielding atomically clean graphene as verified by the high-resolution TEM (HRTEM) image in (figure 4*d*). Quantitative analysis using TiO₂ nanoparticle adsorption revealed a substantial reduction in surface contamination across the metre-scale films (figure 4*e*). This CO₂-based cleaning step significantly enhances the structural purity and application-relevant properties of CVD-grown graphene [86]. Surface-energy-driven grain merging and self-alignment are also important for achieving single crystallinity. Ta *et al.* [87] explained the self-alignment process, in which randomly oriented secondary graphene flakes nucleate atop a polycrystalline graphene underlayer, rotate gradually and align with each other in the same crystallographic direction (figure 4*f*–*i*). Initially, AB-stacked hexagonal flakes are formed with arbitrary orientations; however, long-range strain and surface diffusion gradients drive their progressive rotational alignment. As these flakes grow, they develop fractal-like morphologies that reflect an evolving twist angle relative to the underlying graphene. Ultimately, the aligned flakes merge seamlessly into a continuous, grain-boundary-free, single-crystal graphene overlayer, despite the polycrystalline nature of the base film (figure 4*j*). Dark-field TEM (DF-TEM) grain mapping confirmed that the upper graphene layer attained a uniform lattice orientation independent of the substrate grain structure (figure 4*k*,*l*). Pang *et al.* [88] compared three Si/SiO_x_ substrate configurations: (i) face-up, (ii) face-down, and (iii) a confined sandwich geometry to achieve direct monolayer graphene growth via thermal CVD (figure 4*m*). The SEM images (figure 4*n*–*p*) and Raman full width at half maximum (FWHM) maps (figure 4*q*–*s*) show that only the sandwich configuration provides uniform nucleation and minimal grain boundary density, yielding highly homogeneous monolayer graphene. Confinement between two SiO₂ surfaces enhances the local concentration of carbon precursor and stabilizes surface reactions, enabling self-terminating monolayer growth. The Raman spectra (G, 2D and D bands) further confirm improved crystalline quality and reduced defect density in the confined configuration (figure 4*t*–*v*). Overall, this confinement-based CVD strategy enables scalable transfer-free synthesis of graphene directly on Si/SiO_x_, which is suitable for device integration. Each mechanism mentioned above, whether reducing unwanted nucleation or promoting aligned grain coalescence contributes to the overarching goal of suppressing grain boundaries and achieving a unified crystallographic orientation across the graphene film.

**Figure 4.**
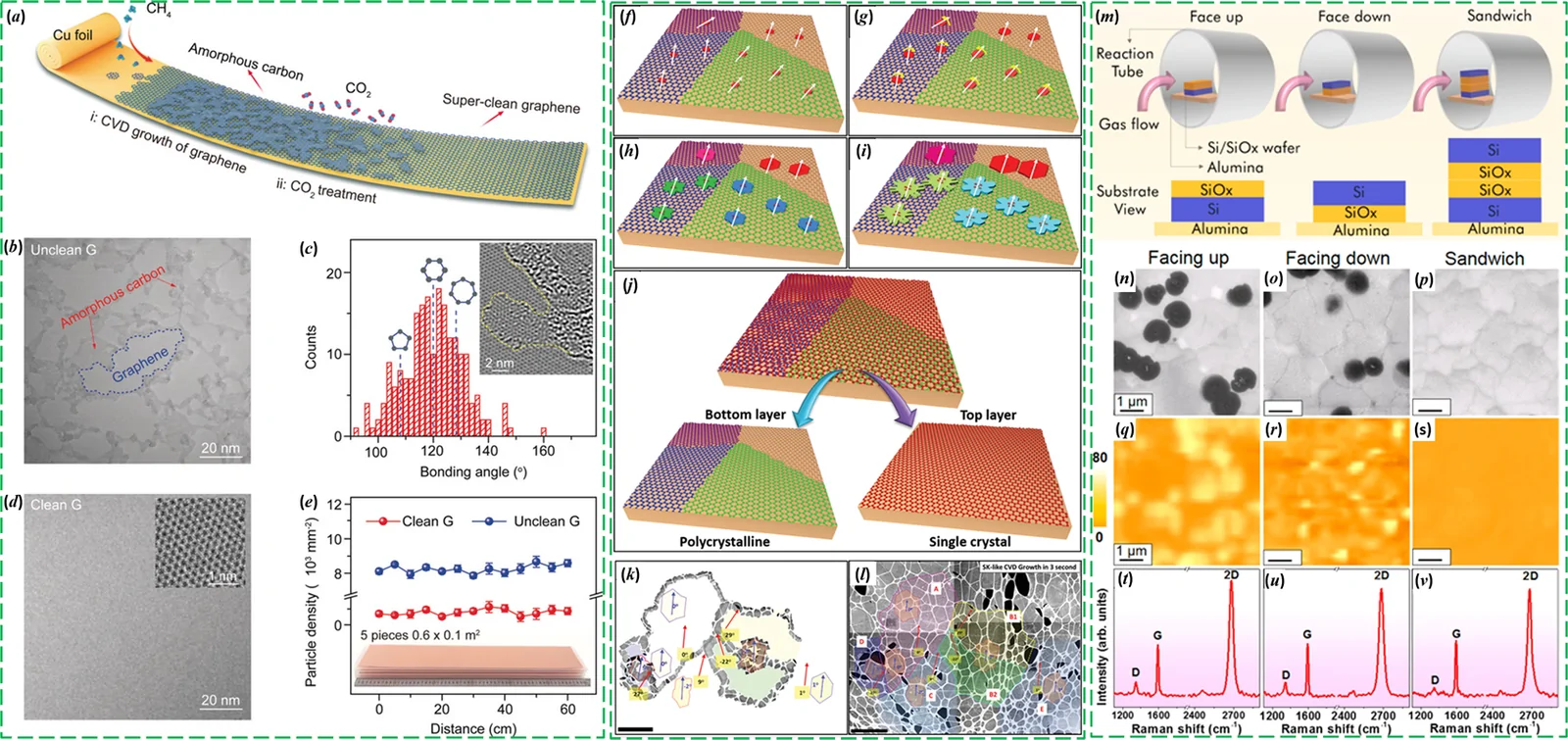
(*a*) Schematic of the formation and elimination of amorphous carbon contamination on the graphene surface during the CVD process. (*b*) Transmission electron microscopy (TEM) image of a typical CVD-grown graphene surface. (*c*) C−C bond angle distribution in amorphous carbon regions after the removal of the underlying graphene lattice using a fast fourier transform (FFT) mask filter. Inset: high-resolution TEM (HRTEM) image of clean and contaminated regions with atomic resolution. (*d*) TEM image of a clean graphene surface prepared using CO_2_ treatment. Inset: HRTEM image of clean graphene with atomic resolution. (*e*) Density of TiO_2_ nanoparticles on clean (red) and unclean (blue) graphene surfaces. Inset: photograph of five 0.6 × 0.1 m sized samples of the clean graphene film after TiO_2_ visualization. (*f*) Schematic showing the growth steps of large-area single-crystal graphene, which is grown from early secondary graphene flakes that self-align over a polycrystalline graphene base. All secondary-layer flakes initially nucleate as randomly oriented AB-stacked hexagons. Reproduced with permission [86]. Copyright 2019, Wiley-VCH. (*g*) Rotation of these flakes to achieve global self-alignment with respect to each other, overriding the underlying polycrystalline graphene orientations. (*h*,*i*) As the twisted flakes grow larger, they acquire more complex (fractal-like) shapes, which strongly correlate with the stacking rotation (twist) angle with respect to the underlying graphene. (*j*) The self-aligned flakes also merge as they grow, yielding a large-area single-crystal graphene domain free of grain boundaries on top of the polycrystalline graphene base. (*k*,*l*) Dark-field TEM (DF-TEM) grain mapping of graphene flakes over a polycrystalline graphene film. The images are stitched together owing to the limited field of view at higher magnifications in TEM. The graphene flakes are oriented/aligned independently of the crystal direction of the underlying graphene film. The red arrows with numbers in yellow boxes indicate the orientation of the underlying graphene film grains with respect to that of grain A. The blue arrows with blue numbers indicate the orientation of a given secondary flake with respect to that of grain A. Scale bar: 5 µm. (*m*) Schematic of substrate configurations explored for the direct synthesis of graphene over Si/SiO_x_ through thermal CVD: SiO_x_ facing up, SiO_x_ facing down and a sandwiched Si/SiO_x_–SiO_x_/Si configuration. The oxide layer is the desired substrate surface for graphene growth. The substrate is a commercially available Si wafer with a polished layer of thermally grown oxide (thickness approx. 300 nm). The supporting plate is sintered alumina. Reproduced with permission [87]. Copyright 2019, Wiley Adv. Portfolio. (*n*–*v*) Characterization of graphene synthesized using the three substrate configurations in (*m*). (*n*,*o*,*p*) SEM images of the post-synthesis sample surfaces. (*q*,*r*,*s*) Raman spectral mapping of the full width at half maximum (FWHM) of the 2D band. Units for the colour scale bar: cm^−1^. (*t*,*u*,*v*) Raman spectra showing the representative G, 2D and D bands of the graphene samples. Reproduced with permission [88]. Copyright 2017, Am. Chem. Soc.

#### 2.1.5. Chemical vapour deposition strategies for direct graphene growth on dielectric and insulating substrates

Recent advances have enabled the direct CVD growth of monolayer graphene on insulating substrates such as oxides, nitrides and two-dimensional materials, addressing the need to avoid transfer steps while maintaining high film quality [89–91]. This shift eliminates the detrimental effects of transfer-induced contamination, positioning insulating substrates as viable platforms for scalable device integration. Graphene directly grown on planar insulators rather than on micrometre- and nanometre-sized powders is readily applicable to various devices, such as graphene field-effect transistors (FETs) [92,93], light-emitting diodes [94] and transparent electronics [95,96]. Numerous CVD techniques have been optimized to achieve this shift, including conventional thermal CVD [97], low-pressure CVD [98] and PECVD [99].

In the absence of metal catalysts, these insulating surfaces exhibit low activity for carbon dissociation; therefore, strategies, such as surface activation and catalytic assistance are crucial [100]. For example, plasma activation generates reactive radicals that decompose methane at lower substrate temperatures, although the reduced surface mobility at low temperatures can increase the nucleation density and yield smaller grains [101]. The first attempts in this direction involved the direct growth of graphene on conventional planar dielectrics, such as SiO_2_/Si, owing to the high availability and device processability of such substrates. In one of the early studies, Chen *et al.* [102] reported the direct deposition of graphene on SiO_2_/Si by atmospheric pressure CVD (APCVD), in which the inert substrate was subjected to oxygen-aided pretreatment to activate it for graphene nucleation. To enhance growth efficiency, Liu *et al.* [37] and Wei *et al.* [103] later applied the water-assisted CVD process to SiO₂/Si (figure 5*Ia*), where trace water vapour acted as a mild oxidant that simultaneously lowered the kinetic barrier for CH₄ decomposition and prevented premature carbon aggregation. This oxidative effect accelerated nucleation, promoted uniform lateral growth and suppressed defect formation, yielding monolayer graphene with higher hole mobility and ON/OFF ratio than those of conventional CVD graphene. Additionally, the growth rate increased because of the lower energy barriers for methane decomposition with oxygen (figure 5*Ib*). Transitioning from planar oxides to crystalline substrates, figure 5*II*. A notable advance in direct CVD growth on insulating substrates is the demonstration of wafer-scale SLG on H₂-etched c-plane sapphire (α-Al₂O₃(0001)), where detailed local characterization revealed that apparent macroscopic uniformity can coexist with important nanoscale interfacial heterogeneity. In this study, the SLG grown by metal-free CVD exhibited two distinct interfacial regimes: G-I, located on hydroxyl-terminated sapphire terraces, where graphene remains weakly but distinctly coupled to the substrate through weak H-bond-mediated interactions and forms a Moiré-like superstructure; and G-II**,** located near sapphire step edges, where local delamination produces quasi-free-standing graphene regions with higher conductivity. As shown in figure 5*II*, AFM/phase/SEM analyses ((a), (b), (d), (e)) identify terrace–step topography and wrinkle formation near step edges, while Raman spectra (c) confirm the SLG character and (f) the schematic summarizes the terrace-bound versus step-edge-delaminated graphene domains. Importantly, the terrace-supported graphene shows a measurable band-gap opening (approx. 73 meV at the Dirac point) owing to charge injection from the substrate, whereas the step-edge regions behave as conductive pathways, highlighting how substrate topography and local interface chemistry band-gap opening (approx. 73 meV at the Dirac point) govern the electronic uniformity of directly grown graphene on sapphire, a key consideration for device-grade dielectric-substrate CVD [104]. Figure 5*III* presents the sequential CVD growth of graphene on hexagonal BN (h-BN) films (which had been grown on Cu foils, figure 5*IIIa*), producing atomically clean single-layer h-BN/graphene heterostructures (figure 5*IIIb*). Scanning tunnelling microscopy (STM) images reveal straight step edges in the h-BN layer, clear Moiré patterns with periods of 0.55 and 4.2 nm (indicating alignment and small misorientation angles), and atomic-resolution images confirm the honeycomb lattice (figure 5*IIIc*–*e*). The cross-sectional TEM images (figure 5*IIIf*,*g*) and electron energy loss spectroscopy (EELS) data (figure 5*IIIh*) verify that the graphene/h-BN stack is robust and transferable to SiO₂. Electrical measurements further show higher carrier mobility and reduced impurity scattering compared with graphene/SiO₂ or transferred graphene/h-BN devices [105], h-BN, which is a structural analogue of graphene, is an insulator with a typical bandgap of 5.8 eV and has high in-plane mechanical strength and good chemical inertness [107]. A recent study exploited a temperature-triggered chemical switching mechanism for the selective CVD growth of in-plane (h-BN-graphene) and vertically stacked (graphene/h-BN) heterostructures on insulating substrates, where CO₂-mediated etching enabled zigzag-edged lateral stitching at high temperatures and van der Waals epitaxial alignment at lower temperatures [108]. The present authors designed a co-segregation approach for the direct growth of graphene/h-BN heterostructures with solid-state dissolved constituents (C, B and N sources) in the bulk metals as precursors. This protocol provides a potential avenue for batch producing graphene/h-BN stacked hybrids [109]. Such graphene/h-BN stacks are prototypical of the wider family of van der Waals heterostructures, whose electronic and optoelectronic behaviour has been reviewed in depth [110]. Glass, as a widely used amorphous dielectric, is another attractive platform. Graphene-coated glass has been envisioned for real-world applications that combine transparency, conductivity and mechanical stability. The coating routes thus far include: solution printing of liquid-exfoliated graphite nanoplatelets [111], transfer printing of CVD-grown graphene from metal substrates [112] and direct CVD coating of graphene on glass. Compared with other approaches, direct CVD is economical, facile, clean and better suited for scalable graphene production. However, synthesizing graphene on glass has only achieved limited success [113,114]. To address this, one research team devised a catalyst-free thermal CVD route targeting well-controlled synthesis of uniform, high-quality graphene on high-temperature-resistant glasses (such as quartz and sapphire glasses), considering that such graphene directly grown on glasses could be used in real heating devices, low-cost electrodes and smart windows [96]. Substrates that possess higher dielectric constants (*κ*) than SiO_2_ (*κ* ≈ 4) are promising for reducing gate leakage and improving gate capacitance, thereby resulting in greater miniaturization [115,116]. Therefore, it is desirable to directly grow graphene on high-*κ* materials, which would in turn spur the development of high-performance graphene electronics. Figure 5*IV* illustrates two-stage metal-catalyst-free CVD on Si₃N₄, involving separate nucleation and growth stages: a short high-temperature step nucleates sparse graphene islands, followed by a lower-temperature growth phase that expands the islands without forming new nuclei. The AFM and TEM images reveal continuous polycrystalline graphene directly grown on Si₃N₄ without a transfer step, and the corresponding FETs demonstrate carrier mobilities of approximately 1.5 × 10³ cm² V⁻¹ s⁻¹, rivalling that of some metal-assisted CVD films. To date, researchers have tested numerous high-*κ* materials to achieve this goal, such as ZrO_2_ [116], Si_3_N_4_ [106], SrTiO_3_ [117] and CuO [118]. Collectively, these examples demonstrate how chemical activation (water vapour), cyclic plasma cleaning, use of heteroepitaxial templates (h-BN) and staged nucleation/growth can overcome the low catalytic activity of oxides, nitrides and two-dimensional dielectrics, enabling the direct growth of high-quality monolayer graphene on non-metal substrates (table 1).

**Figure 5.**
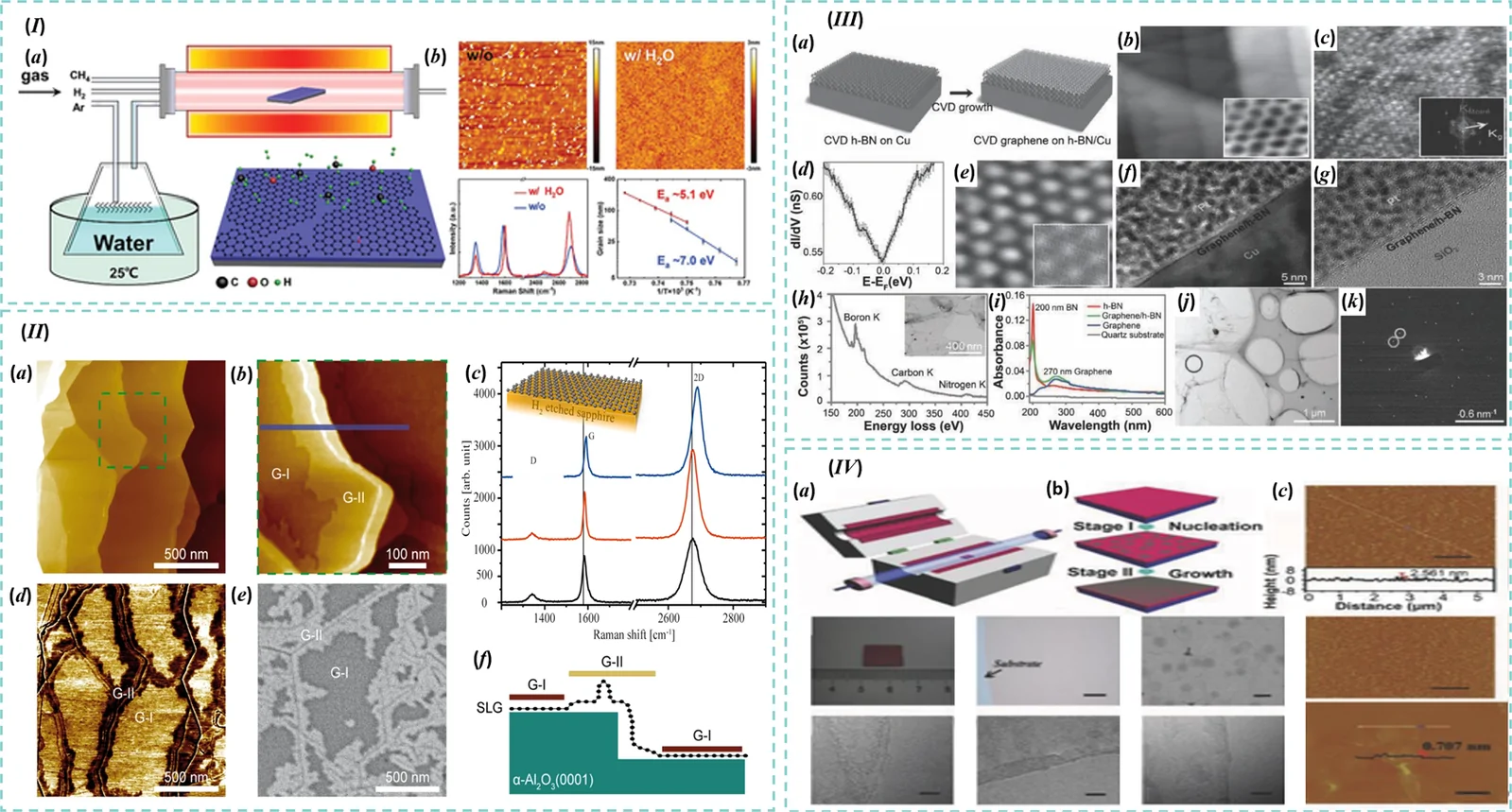
(*I*) (*a*) Schematic of water-assisted CVD growth of graphene on SiO_2_/Si substrate. (*b*) AFM and Raman results of graphene grown with and without water. Reproduced with permission [103]. Copyright 2019, Elsevier. (*II*) AFM and SEM measurements of SLG on α-Al2O3 (0001). (*a*) Large-area AFM image showing the edges of the sapphire steps. (*b*) Higher-resolution image of the area marked in (*a*) (green square) showing an α-Al_2_O_3_ (0001) step and wrinkles near the step edges. (*c*) Raman measurements of SLG on H_2_-etched sapphire. (*d*) Corresponding phase image to (*a*). (*e*) SEM image. (*f*) Schematic of delamination near the step edges of sapphire. Two SLG regions can be seen, one on flat sapphire terraces and the other in the vicinity of sapphire step edges; they are labelled G-I and G-II, respectively, in (*b*)–(*f*) [104]. Copyright 2022, Nature. (*III*) (*a*) Schematic of CVD-grown graphene on the h-BN film/Cu foil. (*b*) STM image with the straight step edges of the underlying h-BN layers. Image area: 60 × 60 nm. Parameters: *V*_b_ = 1.0 V, *I*_t_ = 0.1 nA. Inset: STM image of the graphene honeycomb lattice. (*c*) STM image related to the Moiré pattern with a 0.55 nm period. Inset: FFT result of (*c*). Image area: 10 × 10 nm. Parameters: *V*_b_ = −0.8 V, *I*_t_ = 0.12 nA. (*d*) Average d*I*/d*V* values determined in the region of 0.55 nm Moiré pattern, with the error bars overlaid at each energy point. Parameters: *V*_b_ = 0.2 V, *I*_t_ = 0.2 nA. (*e*) STM image showing the Moiré pattern having a period of 4.2 nm. Image area: 23 × 23 nm. Parameters: *V*_b_ = − 0.14 V, *I*_t_ = 0.11 nA. Inset: simulated Moiré pattern (4.2 nm) having an angle of 3.2° between graphene and h-BN. Cross-sectional TEM images of the (*f*) CVD-grown graphene/h-BN film on the Cu foil and (*g*) CVD-grown graphene/h-BN film on the SiO_2_/Si substrate. (h) Electron energy loss spectroscopy (EELS) spectrum indicating elements B, N and C. Inset: top-view TEM image of graphene/h-BN film. (*i*) UV-visible spectra of h-BN grown by the CVD technique. (*j*) TEM image of a graphene/h-BN film. (*k*) Selected area electron diffraction pattern of graphene/h-BN. Reproduced with permission [105]. Copyright 2013, Wiley-VCH. (*IV*) (*a*) Schematic of the CVD system for growing graphene on a Si_3_N_4_ substrate. (*b*) Schematic of the two-stage process for graphene growth. (*c*) AFM images of the Si_3_N_4_ substrate before and after nucleation, height images. Scale bars: 1 µm, photograph of the graphene film on the Si_3_N_4_ substrate; optical image of a graphene film on a Si_3_N_4_ substrate. Scale bar: 50 µm, SEM image of the graphene film. Scale bar: 500 nm, HRTEM images of graphene films. Scale bars: 5 nm, AFM images of transferred graphene on SiO_2_/Si substrates. Reproduced with permission [106]. Copyright 2013, Wiley-VCH.

**Table 1.** Characteristics of substrates commonly used for growing monolayer graphene by CVD.

substrate	carbon solubility	growth mode	monolayer tendency	key challenges/notes	ref.
polycrystalline Cu	*very low* (∼10^−5^ at.% at 1000°C)	surface-only (no bulk C uptake)	Strong self-limited monolayer growth, but the grown graphene is polycrystalline.	Many nucleation sites (grain boundaries and roughness) indicate smaller domains; it requires polishing/annealing to reduce multi-layer patches.	[39,57,62]
Cu(111) single-crystal	*very low* (same as poly Cu)	surface-only, epitaxial alignment	Excellent monolayer uniformity, single-crystal graphene is achievable.	It practically eliminates multi-layers; large single-domain graphene (cm-scale) is demonstrated; high cost or effort is required to prepare single-crystal Cu.	[57,62]
Ni (poly or single)	*high* (0.1–1 at %)	bulk dissolution + surface segregation during cooling	Tends to form multi-layer graphene (uncontrolled layer number).	It is difficult to get strictly monolayer because carbon precipitates upon cooling down. Rapid quench can limit layers but often yields patchy coverage.	[39,62]
Cu-Ni alloys	*intermediate* (tuneable by Ni%)	primarily surface (if Cu-rich), slight bulk absorption	Can achieve near-monolayer with optimized Ni content and cooling.	Ni enhances CH₄ cracking (faster growth), while Cu limits excess carbon. Carbon precipitation on cooling must be prevented.	[51,52]
Pt, Ir or Ru single-crystal	*very low* (negligible)	surface-only, strong chemisorption	Forms monolayer graphene that often has high crystalline quality.	Graphene strongly binds to the metal, impeding transfer. Substrates are expensive. The method is typically used in research rather than scalable production.	[34,112]
Ge(001) or Ge(110)	*negligible* (no carbide formation)	surface-mediated (catalyst-free)	Monolayer possible on wafer-scale, polycrystalline film with multiple orientations.	It allows direct growth on the semiconductor wafer. Domain alignment and grain boundaries are remaining issues. Graphene can be transferred by etching or picked up owing to weak binding to Ge.	[76]
dielectrics (e.g. Si₃N₄)	non-catalytic	requires activation (plasma or metal film)	Direct monolayer growth typically yields graphene with many defects or multi-layer graphene, unless aided by metal catalysts.	A new method that uses sacrificial metal films or plasma to achieve graphene on insulators. Graphene is usually polycrystalline and contains small grains.	[106]
h-BN on Cu (bilayer substrate)	h-BN is inert, Cu beneath has low carbon solubility	surface growth on h-BN is enabled by Cu underneath	Monolayer graphene can grow atop an h-BN film of clean interface graphene/h-BN stack.	The h-BN layer (≤3 nm) on Cu is ‘catalytically transparent’, allowing the formation of large-area graphene on h-BN by CVD. Graphene inherits the smoothness of h-BN. After graphene growth, Cu is etched to transfer the heterostructure.	[105]

### 2.2. Epitaxial Growth on SiC

Epitaxy is a technique for growing single-crystalline films on crystallographically oriented crystalline substrates following crystallographic registration of the substrate [119]. Epitaxial growth of graphene on SiC substrates occurs by thermal decomposition at high temperatures, generally above 1200°C [120]. At this temperature, Si atoms evaporate from the SiC surface, leaving behind C atoms that form the graphene structure [121]. This process is often performed in an inert gas atmosphere (such as Ar) to control the sublimation rate and improve the uniformity of the graphene layer [122]. One unique advantage of this method is that it requires no transfer of graphene to other substrates, thereby reducing the risks of contamination and mechanical damage [123]. During the epitaxial growth of graphene on SiC, several important factors significantly affect the quality and characteristics of the product.

#### 2.2.1. Step-flow growth dynamics

Graphene formation on SiC is strongly influenced by the substrate morphology. The SiC wafers are usually cut slightly off-axis, yielding atomic steps on the surface. During annealing, Si sublimation is preferentially initiated at these step edges where binding is weaker, and C atoms released from the receding steps nucleate to form graphene along the step edge [124]. In a step-flow growth mode, graphene extends from the steps onto the terraces like a carpet. Each decomposed SiC bilayer leaves C atoms behind to enlarge the graphene layer. Indeed, C atoms from roughly one SiC bilayer can cover a terrace area between the steps [124]. This often leads to step bunching, in which smaller steps merge into higher steps to create wider terraces covered by monolayer graphene. Under optimal conditions, the graphene film remains continuous across these step bunches. For example, with controlled sublimation, one can obtain micrometres-wide terraces covered by SLG that seamlessly cross the step edges [124]. In a recent breakthrough, the control over this dynamics was improved using a kinetics-controlled thermal shock annealing (TSA) method, enabling the formation of high-quality graphene within just 5–10 s in Ar at ambient pressure and approximately 1800°C [125]. As illustrated in figure 6*a*–*e*, this ultrafast annealing process significantly outperforms conventional vacuum and furnace annealing in terms of both time and energy efficiency. Noticeably, it mitigates the step-bunching phenomena typically observed on SiC terraces. The Raman spectra (figure 6*f*) confirm the formation of monolayer-to-bilayer graphene with a minimal D-band intensity, indicating negligible structural defects. AFM images (figure 6*g*) also show that the epitaxial growth on SiC via TSA results in a substantially reduced surface roughness (approx. 0.28 nm) in relation to that of furnace-grown films (approx. 7.5 nm), highlighting improved flatness that is critical for electronic and photonic integration. The TSA method positions itself favourably in the quality-cost-performance landscape of graphene synthesis (figure 6*h*). It offers a scalable and potentially automated pathway (figure 6*i*) for producing wafer-scale epitaxial graphene (EG) with real-time Raman-based quality control. This ability to rapidly and reproducibly synthesize mono/bilayer graphene on both α- and β-SiC platforms marks a significant step toward practical applications in surface-enhanced infrared absorption sensing, quantum metrology and high-frequency electronics [125].

**Figure 6.**
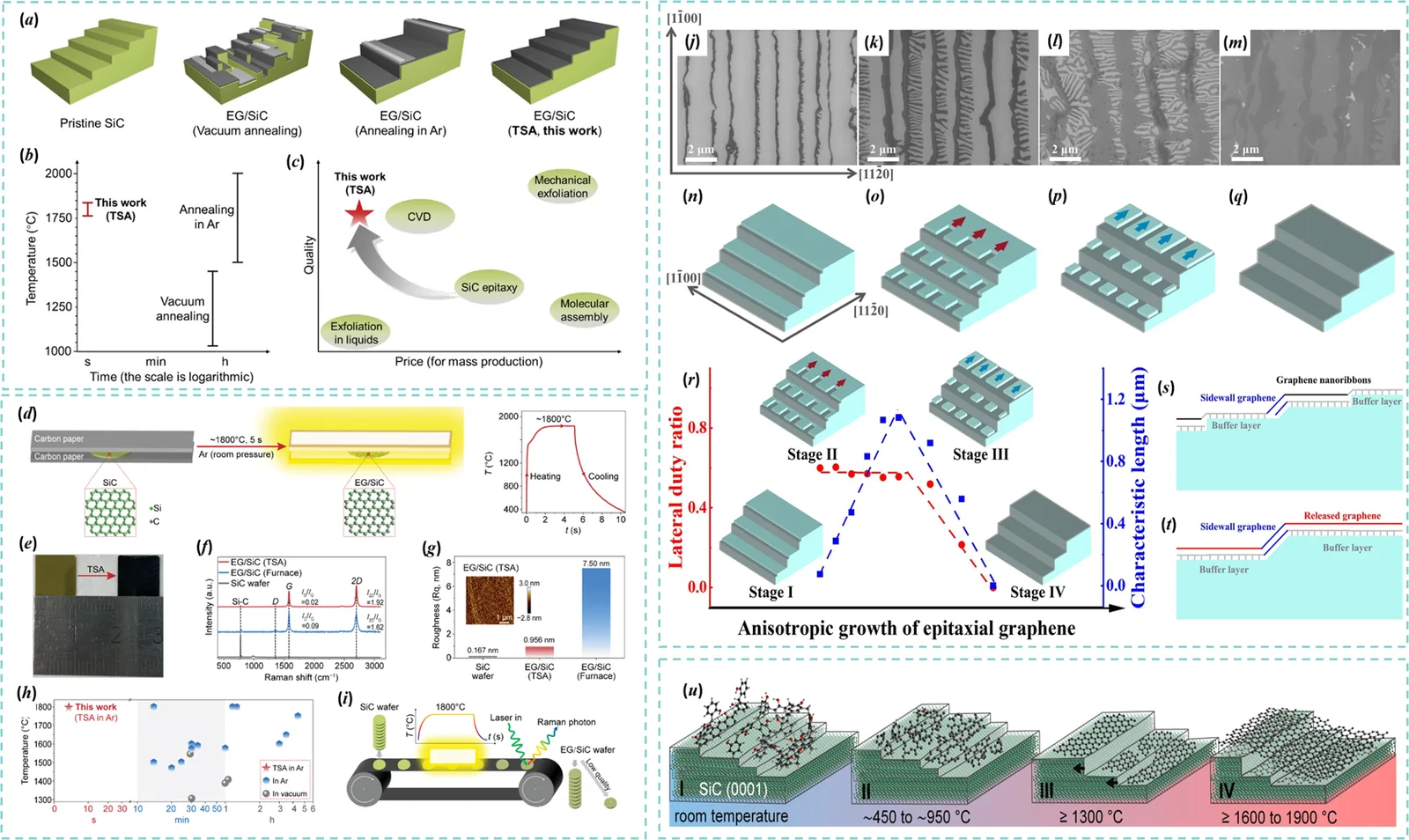
(*a*) Evolution of the SiC surface during the epitaxial growth of graphene (EG: epitaxially grown graphene). (*b*) Comparison between TSA and other reported methods of growing EG on SiC, indicating the high time and energy efficiency of the TSA method. The error bar shows the applied temperature window for EG. (*c*) Price versus quality for the typical methods to mass-produce graphene. The TSA method is expected to yield higher quality at a lower price, thus offering competitive advantages for practical applications. Reproduced with permission [125]. Copyright 2023, Wiley Adv. Portfolio. (*d*) Schematic of the growth of EG on the SiC wafer, along with temperature evolution during this process. (*e*) Photograph of the raw square-centimetre-sized SiC wafer and derived EG/SiC wafer. (*f*) Raman spectra and (*g*) surface roughness of the SiC wafer, EG/SiC wafer (TSA) and EG/SiC wafer (furnace). Inset in (*g*) AFM image of the EG/SiC wafer (TSA). (*h*) Time and temperature employed for EG growth in this work and in the literature. (*i*) Schematic for the continuous and automatic synthesis of the EG/SiC wafer. Real-time and online monitoring systems equipped with Raman spectrometers will help with the quality control of EG/SiC. Reproduced with permission [125]. Copyright 2023, Wiley Adv. Portfolio. (*j*–*m*) SEM images of the SiC surface after heating for 54, 60, 64 and 76 min. The light grey regions are the buffer layer, and the dark grey regions indicate graphene. (*n*–*q*) Schematic of EG formation at different growth stages. Red and blue arrows show the growth directions. (*r*) Different stages of anisotropic growth of EG. (*s*,*t*) Schematic of graphene structures in different growth stages. Reproduced with permission [126]. Copyright 2025, Elsevier. (*u*) General schematic of the PASG of graphene on SiC(0001) in four consecutive steps. (I) Deposition of the polymer adsorbate. (II) Thermal decomposition and cross-linking of the adsorbate. (III) Conversion of deposited carbon to the buffer layer. (IV) Continuous silicon sublimation and transformation to graphene. Reproduced with permission [127]. Copyright 2016, IOP Sci.

Beyond rapid annealing, researchers recently gained deep mechanistic insights into the epitaxial growth of graphene on 4H-SiC(0001) through the detailed investigation of polymer-assisted sublimation growth (PASG) under low-pressure conditions. As illustrated in figure 6*j*–*t*, graphene formation in this case progresses through four distinct stages: deposition and thermal decomposition of a polymeric carbon source (stages I and II), carbon incorporation into a buffer layer (stage III) and transformation into monolayer graphene upon silicon sublimation (stage IV). The SEM images (figure 6*j*–*m*) reveal a dynamic evolution of surface morphology with the heating time, transitioning from narrow, isolated graphene nanoribbons (GNRs) to fully merged monolayers. The corresponding schematics (figure 6*n*–*q*) depict growth driven by step-edge-guided anisotropic kinetics on SiC terraces, which is attributed to varying sublimation kinetics across the surface. Quantitative analysis shows that the GNRs have widths of 40–300 nm and a maximum length exceeding 1 µm, emphasizing the role of silicon sublimation rate in driving anisotropic ribbon elongation (figure 6*r*). The anisotropic growth mechanism is further clarified by structural models of graphene progression along terraces (figure 6*s*,*t*), where sidewall graphene and buffer interfaces evolve into extended nanoribbon domains [126]. Notably, the general schematic (figure 6*u*) outlines the PASG sequence from polymer deposition to thermally activated conversion to high-quality graphene. This study establishes that the silicon sublimation rate, which is governed by the local silicon atom concentration under quasi-equilibrium, plays a pivotal role in directing the lateral growth dynamics of GNRs. These findings advance the understanding of EG formation on SiC and offer guidance for achieving large-area, uniform monolayer graphene through controlled anisotropic kinetics [127]. They inform the future integration of graphene into electronic and photonic devices.

Over the past decade, the quality of SiC-grown graphene has improved significantly and become more suitable for industrial applications. The major focuses are increasing the lateral domain size and achieving more uniform monolayers. By using controlled-atmosphere furnaces (Ar ambient or confined sublimation methods), researchers have grown millimetre-scale single-crystal domains of monolayer graphene on the Si-face. For example, Yazdi *et al.* [124] reported homogeneous monolayer domains up to 2 × 2 mm on the Si-face of 4H-SiC under optimized high-temperature Ar sublimation conditions. Those large domains were practically continuous over the entire wafer, drastically reducing the grain boundaries. To further advance domain uniformity and interface control, the confinement-controlled sublimation (CCS) approach developed at Georgia Tech (i.e. encapsulating SiC in a graphite enclosure to retain Si vapour) has been a game changer that allows near-equilibrium growth and produces very high-quality graphene on both faces. Multi-layer graphene grown using CCS on the C-face was found to consist of many rotationally faulted but electronically decoupled layers, functionally behaving like isolated monolayers with high carrier mobility. This finding relaxes the demand for strictly monolayer films in some applications because even a stack of several twisted layers can electrically behave like independent monolayers [123].

In the pursuit of high thermal efficiency and rapid growth, researchers proposed a hybrid approach called inner and external carbon synergy (IECS) to combine the advantages of CVD and conventional epitaxial growth on 6H-SiC, achieving high-quality graphene synthesis with reduced time and temperature constraints [128]. As depicted in figure 7*a*–*d*, hydrogen etching forms atomically ordered terraces, followed by methane-assisted growth at elevated temperatures to preferentially form graphene on the Si-face. Remarkably, the IECS method enabled monolayer graphene growth within just 3 min at an optimized temperature of 1450°C, approximately 200°C lower than that of traditional epitaxial protocols. In the Raman spectra (figure 7*b*,*c*), graphene grown under the optimal conditions (CH₄ flow rate: 1.5 sccm, 1450°C) exhibits a high I_2D_/I_G_ ratio and a minimal D band, which are hallmarks of low-defect, monolayer graphene. Surface morphology captured in the AFM images (figure 7*d*,*f*) shows continuous terraces post-etching and uniform graphene coverage after growth, while the Raman analysis and X-ray photoelectron (XP) spectroscopy (XPS) (figure 7*e*,*g*–*i*) confirm the monolayer character and effective interface formation. This IECS strategy significantly enhances the scalability of SiC-based graphene synthesis and improves the compatibility with complementary metal-oxide-semiconductor, thus offering a thermally efficient, transfer-free route to high-quality graphene directly grown on semiconductor-grade substrates suitable for microelectronic integration [128].

**Figure 7.**
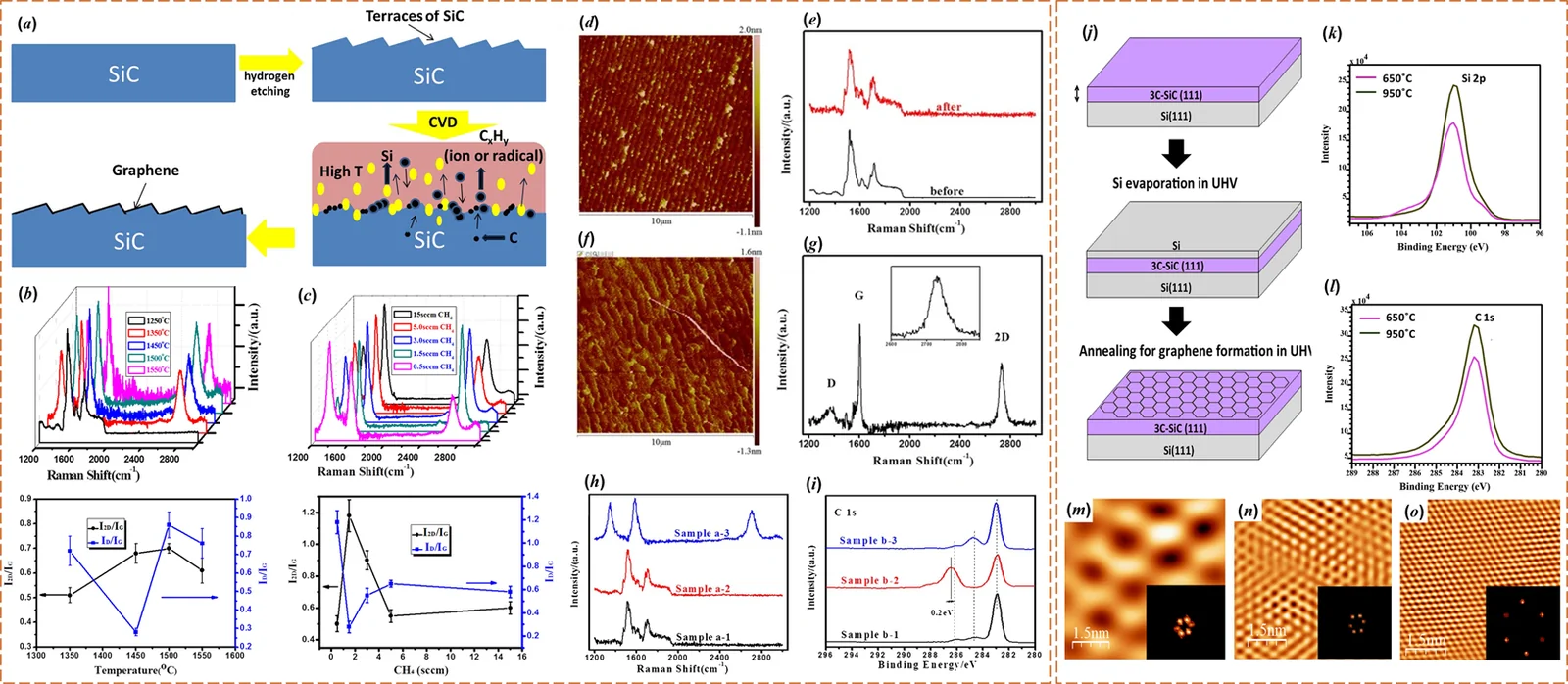
(*a*) Schematic of the basic proposed approach for directly growing high-quality graphene on the 6H-SiC substrate. The illustrations show cleaning of the substrate, well-ordered atomic terraces after hydrogen etching and preferential formation of the graphene single layer on the substrate surface. (*b*) Typical processed Raman scattering spectra of graphene (top) and I_2D_/I_G_ and I_D_/I_G_ ratios (bottom) at different growth temperatures. Fixed growth conditions: 5 min, methane flow rate of 1.5 sccm. (*c*) Typical processed Raman scattering spectra of graphene (top) and I_2D_/I_G_ and I_D_/I_G_ ratios (bottom) at different methane flow rates. Fixed growth conditions: 1450°C, 3 min. (*d*) Typical AFM image of the 6H-SiC substrate after hydrogen etching at 1550°C for 20 min. (*e*) Raman spectra of the 6H-SiC substrate before and after hydrogen etching. (*f*) AFM image of the graphene film grown at 1450°C for 3 min. (*g*) Raman spectra of the obtained graphene film after subtracting SiC contribution. (*h*) Raman spectra and (*i*) C 1s X-ray photoelectron (XP) spectra of the three differently prepared samples. Reproduced with permission [128]. Copyright 2018, Elsevier. (*j*) Schematic diagram of graphene epitaxial growth process. XP spectra of the Si-rich surface SiC(111) after annealing at 650 and 950°C with Si flux: (*k*) Si 2*p* and (*l*) C 1*s* regions. (*m*–*o*) Moiré patterns from (*m*) the innermost hexagon, (*n*) second-nearest neighbours from the middle hexagon and (*o*) first-nearest neighbours (in the three-for-six symmetry of graphene atoms) from the outer hexagon. Reproduced with permission [129]. Copyright 2014, Elsevier.

Complementary to the IECS method, an emerging cost-effective alternative to bulk SiC substrates is integrating high-quality graphene with Si-compatible platforms and epitaxial growth on 3C-SiC(111)/Si(111) heterostructures [129]. In this approach, a 3C-SiC layer is first deposited on Si(111), followed by high-temperature annealing under ultra-high vacuum (UHV) to drive Si sublimation and promote graphene formation at the surface (figure 7*j*). XPS analysis of the annealed surfaces revealed clear temperature-dependent shifts in the Si 2*p* and C 1*s* spectra (figure 7*k*–*l*). Higher annealing temperatures (up to 1375°C) are correlated with stronger C–C bonding and more complete Si desorption, consistent with increased graphene coverage. The atomic-scale STM images (figure 7*m*–o) show distinct Moiré patterns and lattice symmetries, confirming the presence of monolayer and FLG with various stacking configurations. Although elevated temperatures improve the crystalline quality (as evidenced by the reduced Raman D-band intensity), they also increase the layer thickness, creating a trade-off for applications that require strict monolayer uniformity. Specifically, there is an Arrhenius-type dependence of graphene layer number on the annealing temperature, with monolayer formation achievable at approximately 1200°C and multi-layer growth (up to eight layers) at higher thermal budgets. Although elevated annealing enhances the layer quality according to the reduced Raman D-band intensity, it simultan eously increases the layer thickness, thereby posing a trade-off for device-oriented applications. Despite this trade-off, the direct growth of graphene on Si-compatible substrates using 3C-SiC(111)/Si(111) paves the way for the monolithic integration of graphene-based electronic and optoelectronic platforms via a UHV-compatible, transfer-free process [129].

### 2.3. Arc discharge method

The arc discharge method uses electrical discharge between graphite electrodes in a gaseous environment to form a carbon plasma, which condenses into graphene, usually FLG. Typically, a DC arc (tens of amperes, 30–120 V) is struck between pure graphite rods (5–10 mm in diameter) in an inert or redu cing gas at approximately 0.5–1 atm [130]. This process can produce high-quality graphene with a good crystalline structure. However, variables, such as gas pressure and current, have to be controlled exactly to guarantee homogeneity and purity [131]. Although large-scale production is possible with this straightforward process, minimizing contamination and flaws is a challenge [132]. The process also critically depends on parameters, such as electrode geometry and composition (anode/cathode materials and catalyst additives), discharge current/voltage, inter-electrode gap, type and pressure of buffer gas and reactor configuration including magnetic fields or cooling mechanisms [25]. For example, Wang *et al.* [133] used a high-current He/H₂ arc (approx. 0.5 atm He), a Y-Ni powder in the anode to improve carbon evaporation and even an optional approximately 0.1 T magnetic field to focus the plasma. The choice of gas was reported to have a strong effect on the layer number: helium favoured monolayer-rich graphene (although the absolute yield was low), hydrogen yielded FLG (approx. 2–4 layers) with minimal nanotube byproducts and argon tended to produce somewhat thicker flakes (approx. 4 layers) [21]. Mixed gases (H₂/N₂) can balance quality and scalability. Borand *et al.* [25] reported that smaller reactor chambers produced thinner flakes, and that high-purity FLG was deposited on the hot anode side where temperature gradients favoured graphene nucleation.

Most arc methods yielded FLG. For example, the arc-produced flakes are typically 3–10 layers thick (lateral size: approx. 1–10 µm) [134]. True monolayers are rare (often less than 1% of flakes), although modelling suggests that higher substrate temperatures lead to larger (approx. 5 µm) graphene sheets with lower adatom density because these conditions suppress additional layer nucleation and favour single-layer growth [134]. The aqueous-arc variations have demonstrated the controlled formation of bilayer and trilayer graphene by exploiting water cooling and turbulence. The gas-dependent variations are particularly important for tailoring the characteristics of graphene for specific applications, such as high mobility, transparency and thermal conductivity. Hybrid methods have been developed to address some limitations of traditional arc discharge, such as the lack of layer control and high thermal input [135]. Figure 8*b*–e compares the morphology and composition of graphene flakes synthesized in Ar and He plasma environments. The scanning transmission electron microscope (STEM) images show that the Ar plasma produces thicker, highly wrinkled and defective multi-layer flakes, whereas the He plasma predominantly yields thin, few-layer to monolayer graphene with a smoother morphology (figure 8*d*,*e*). Energy-dispersive X-ray (EDAX) analysis (figure 8*c*, inset) confirmed comparable carbon purity between the two atmospheres, whereas dynamic light scattering (DLS) data indicated a narrower flake size distribution for graphene grown in He. The superior structural quality and reduced defect density associated with the He plasma are directly correlated with this plasma's higher electrical conductivity, underscoring the advantages of the low-ion-density plasma for bottom-up graphene synthesis [136].

**Figure 8.**
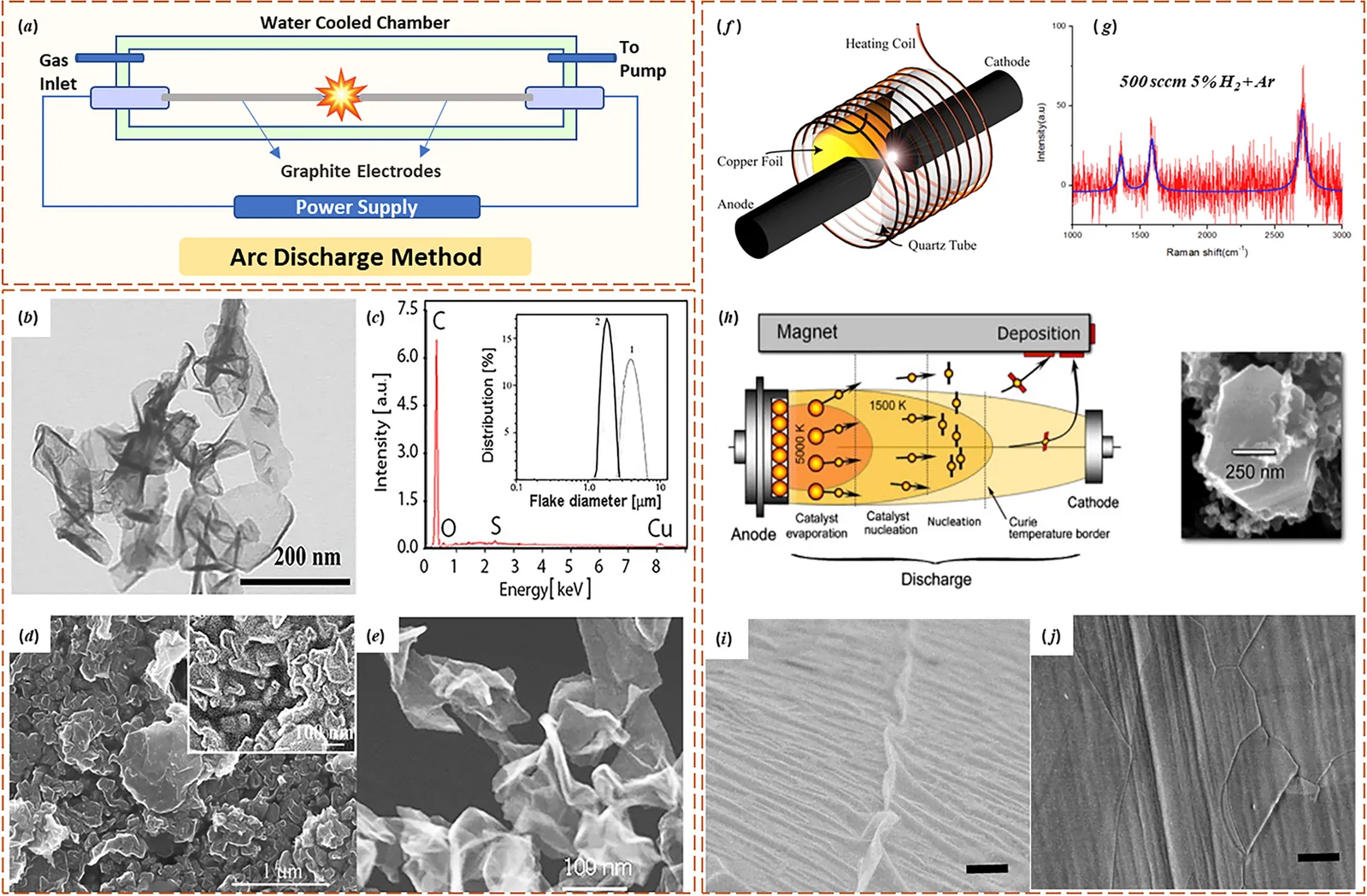
(*a*) Schematic of the arc discharge method. (*b*) Scanning transmission electron microscope (STEM) image showing the morphology of graphene flakes synthesized in the helium plasma. (*c*) Carbon content of as-synthesized graphene determined by EDAX. Inset: distribution of flake diameters, which were measured in liquid ethanol medium by DLS: (1) C-Ar and (2) C-He. (*d*,*e*) SEM images showing the morphology of graphene flakes synthesized in the (*d*) argon plasma and (*e*) helium plasma. Inset in (*d*): magnified image. Reproduced with permission [136]. Copyright 2020, MDPI. (*f*) Schematic of the thermal CVD-embedded vacuum arc discharge system for growing SLG. (*g*) Raman spectrum of graphene grown on Cu in 500 sccm 5% H_2_ + Ar. The high I_G_/I_D_ ratio indicates a high quality of the produced graphene. Reproduced with permission [137]. Copyright 2017, Elsevier. (*h*) Scheme of graphene nucleation, growth and deposition in the magnetic field-enhanced arc discharge. Catalyst particles evaporate in a hot (5000 K) plasma, then nucleate into small (4 nm on average) particles at a lower temperature; further, graphene nucleation starts, along with electron micrograph of a typical carbon nanoparticle containing graphene flakes [138]. Copyright 2010, Elsevier. (*i*,*j*) Field-emission SEM (FESEM) images of graphene synthesized under various processing conditions: (*i*) arc + CVD with vacuum cooling and (*j*) arc + CVD with fast cooling in high-flow Ar. In (*i*), wrinkled graphene films were often observed, the image was taken with the sample tilted at 30° and the scale bar = 10 µm. In (*j*), scale bar = 100 µm. Reproduced with permission [137]. Copyright 2017, Elsevier.

In a promising hybrid approach, the integration of thermal CVD with vacuum arc discharge enables the efficient fabrication of monolayer graphene at reduced substrate temperatures. Here, a graphite-based vacuum arc provides both carbon atoms and rapid heating through photon radiation, elevating the surface temperature of Cu substrates to approximately 1100°C while maintaining a base furnace temperature as low as 600°C [137]. In the embedded thermal CVD set-up illustrated in figure 8*f*, the graphite anode and cathode are enclosed within a quartz tube, allowing localized thermal enhancement via the arc plasma. Optimization studies revealed that a gas mixture of 500 sccm H₂/Ar (5% H₂) ambient atmosphere, controlled arc current and rapid cooling conditions are crucial for producing uniform SLG films without requiring post-annealing. The Raman spectrum (figure 8*g*) shows a high I_2D_/I_G_ ratio with minimal D-band intensity, indicating high crystallinity of the grown films [137]. A notable magnetically enhanced arc discharge synthesis, where a non-uniform magnetic field controls plasma growth/deposition zones to promote graphene flake formation and collection. In this process, Y-Ni catalyst particles undergo a magnetic-state transition during plasma transport, guiding catalyst-assisted nucleation into a defined collection region and enabling large-scale production of high-quality graphene flakes in an environmentally benign plasma process. As illustrated in the corresponding (figure 8*h*), the proposed mechanism involves catalyst evaporation in the hot arc plasma (approx. 5000 K), subsequent nucleation into small nanoparticles (approx. 4 nm average) at lower temperature and then graphene nucleation/growth on these particles prior to deposition, supporting a plasma-assisted nucleation-and-growth pathway for arc-derived graphene [138]. Field-emission SEM (FESEM) (figure 8*i*–*j*) supports a morphology of continuous wrinkled sheets typical of monolayer graphene on Cu, particularly under vacuum-quenching conditions that minimize amorphous carbon precipitation. This hybrid arc-CVD strategy eliminates the need for toxic gaseous precursors or post-growth annealing while lowering the thermal budget, thereby offering key advantages in cost, simplicity and process scalability. However, challenges remain in the precise control of layer number and scalability of uniform monolayer domains, particularly in transient arc plasma environments. Continued refinement of arc stability, substrate cooling rates and gas-phase dynamics is essential for realizing the full potential of arc-assisted synthesis routes for wafer-scale device-grade graphene [137].

### 2.4. Molecular assembly method

Molecular assembly constructs graphene atom by atom by coupling the designed organic precursors into extended carbon networks, rather than exfoliating bulk graphite. One route is the thermal graphitization (pyrolysis) of preorganized molecular films [139]. Aromatic self-assembled monolayers (SAMs) on metal substrates can be cross-linked (e.g. by low-energy electron irradiation) to form carbon nanomembranes, which are then annealed at high temperatures to yield nanocrystalline SLG [140]. By choosing different precursor molecules and annealing conditions, one can tune the crystallinity and pore structure of the graphene sheet. Neumann *et al.* [141] showed that SAMs containing pyridine or pyrrole units were converted to graphene as nitrogen was expelled during pyrolysis, leaving a percolated graphene network with tuneable nanopore density. Similarly, in solution-phase bottom-up approaches, small aromatic molecules or polymers can be polymerized and pyrolysed to form graphene-like sheets. For instance, simple organics, such as trisodium citrate, have been thermally decomposed to produce ultra-small (approx. 1.3 nm) monolayer graphene quantum dots (GQDs) [142]. In general, these solution-phase methods rely on high temperatures (often several hundred degrees Celsius) and sometimes metal catalysts (Cu, Ni, etc.) to achieve *sp²* carbon fusion. They offer precise control over edge functionalization (through molecular design) but typically yield limited domain sizes or porous films because complete long-range order is difficult to attain in bulk synthesis. In a previous study, nanocrystalline monolayer graphene was fabricated by the electron-induced cross-linking of aromatic SAMs followed by vacuum annealing. This process yielded free-standing graphene sheets with tuneable electrical properties, including insulator-to-metal transition, making them suitable for various substrate types and scalable device applications [140]. The molecular assembly strategy is illustrated in figure 9*a*, where an aromatic SAM is cross-linked by electron irradiation and then either annealed *in situ* or transferred and annealed to form a nanocrystalline graphene film. The inset depicts a Hall-bar device fabricated from such a film [140].

**Figure 9.**
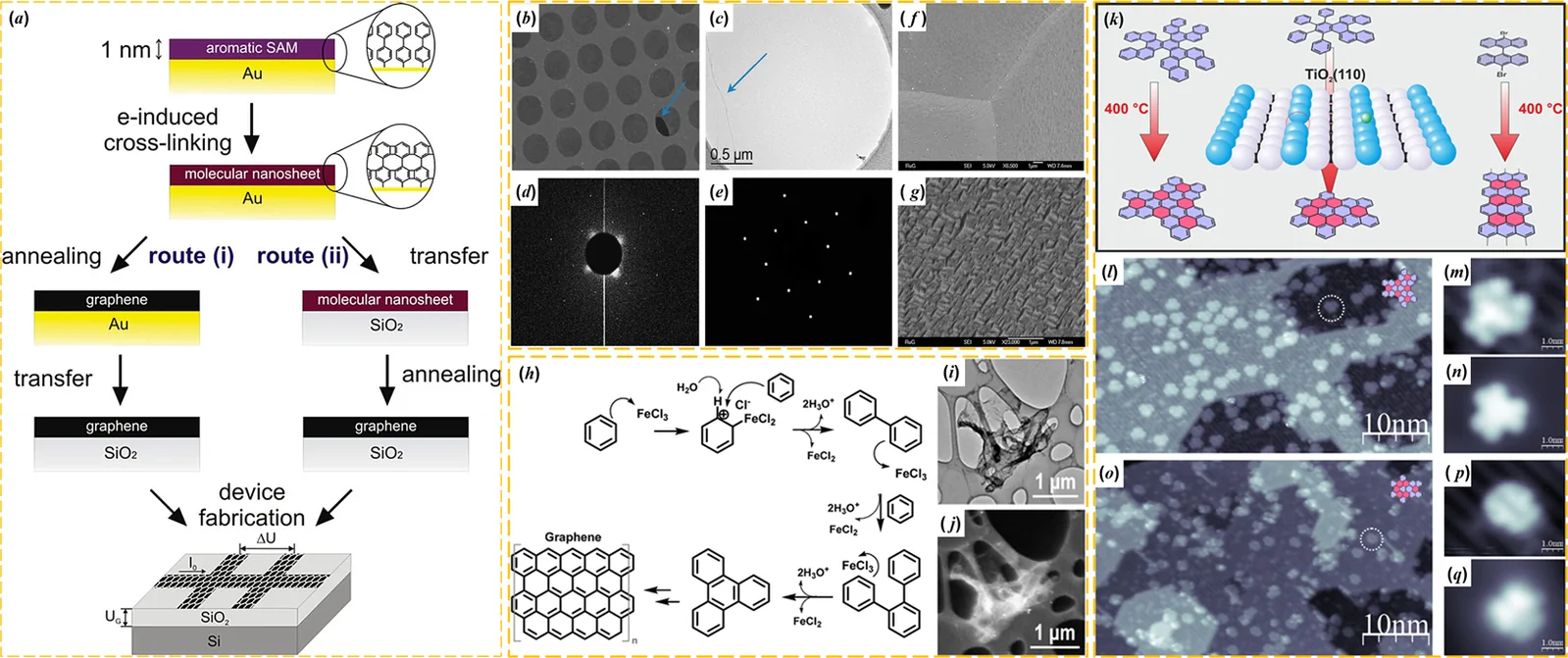
(*a*) Schematic of two routes for fabricating graphene from aromatic SAMs. Reproduced with permission [140]. Copyright 2011, Am. Chem. Soc. (*b*) Overview image of the TEM grid in an area of approximately 250 µm^2^. (*c*) Detailed image of a single layer of suspended graphene. Electron diffraction patterns obtained on the area in panel (*c*), both (*d*) before and (*e*) after background subtraction. (*f*,*g*) SEM images of graphene grown by annealing a photopolymerized biphenylthiol SAM on oxidized Cu. Reproduced with permission [143]. Copyright 2024, Frontiers. (*h*) Mechanism for the conversion of benzene to graphene. (*i*) TEM images of sample GR-BZ and (*j*) the corresponding DF-TEM image. Reproduced with permission [134]. Copyright 2018, R. Soc. Chem. (*k*) Schematic of the formation of two types of nanographene from custom-designed and synthesized molecular precursors on the semiconducting TiO_2_(110)-(1 × 1) surface. Empty-state STM images of (*l*) nanographene and (*o*) nanographene synthesized through cyclodehydrogenation on the TiO_2_(110) surface. Dashed white circles mark exemplary nanographenes. High-resolution STM images of nanographene shown in (*l*) on (*m*) TiO_2_(110) and (*n*) Au(111) and those shown in (*o*) on (*p*) TiO_2_(110) and (*q*) Au(111). Bias voltage: +1.2 V (*l*,*o*,*m*,*p*) or −1.0 V (*n*,*q*). Tunnelling current: 25 pA. Reproduced with permission [144]. Copyright 2023, Am. Chem. Soc.

Recently, a UV-assisted method was used to synthesize monolayer graphene from photopolymerized biphenylthiol SAMs on Cu substrates, followed by vacuum annealing. Superior graphene quality was achieved on oxidized Cu surfaces, highlighting the critical role of substrate properties in SAM-to-graphene conversion [143]. The TEM and electron diffraction data indicate the high structural quality of these films (figure 9*b*–*e*). The suspended monolayer graphene membranes cover an area of approximately 250 µm², with sharp diffraction patterns reflecting their nanocrystalline order. The influence of the substrate microstructure is highlighted in SEM images (figure 9*f*–*g*): graphene grown by annealing a photopolymerized biphenylthiol SAM on oxidized Cu consists of continuous, uniform layers, whereas electropolished Cu yields patchier coverage. These results emphasize the effects of both precursor chemistry and substrate selection on the film continuity and quality [143]. Lopes *et al.* [134] reported a simple chemical route under ambient conditions for synthesizing micrometre-scale graphene sheets at a water/oil interface, using benzene or *n*-hexane with FeCl₃ as an oxidant. This method enables direct film formation via aromatic condensation, bypasses transfer steps and can be extended to prepare doped graphene from heteroatom-containing precursors, such as pyridine (figure 9*h*–*j*). Figure 9*h* outlines the proposed mechanism: benzene is oxidatively coupled to biphenyl and further condensed through electrophilic aromatic substitution to form extended polyaromatic frameworks that ultimately assemble into graphene sheets. The TEM images (figure 9*i*) and the corresponding dark-field images (figure 9*j*) reveal micrometre-sized graphene domains with relatively uniform contrast, confirming that the interfacial reaction can yield sizeable monolayer sheets under mild conditions.

A recent study demonstrated the on-surface synthesis of nanographenes and 7-armchair GNRs on TiO₂(110) via thermally induced intramolecular cyclodehydrogenation of custom molecular precursors [144]. High-resolution STM/scanning tunnelling spectroscopy (STS) and theoretical analyses confirmed precise planarization and structural control, demonstrating the potential of semiconducting surfaces for atomically defined graphene architectures. Figure 9*k* summarizes the approach: planar pentahelicene or dibromobianthryl is deposited on a TiO₂ surface and thermally induced to undergo intramolecular cyclodehydrogenation (and in some cases Ullmann polymerization) to form nanographenes and armchair GNRs. Large-area STM images (figure 9*l*,*o*) show the distributions of the resulting nanographene on TiO₂(110), with white circles highlighting the representative islands. Atomic-resolution STM images (figure 9*m*,*n*,*p*,*q*) compare the nanographene structures formed on TiO₂ and Au(111) substrates, revealing that the planar polycyclic networks retain their intrinsic frontier orbitals and exhibit minimal electronic perturbation by the underlying semiconductor. Together, these results emphasize that rational precursor design and surface-assisted reactions enable atomically precise graphene architectures with controlled sizes and edge structures on semiconducting platforms [144].

An alternative strategy involves on-surface synthesis under UHV to build graphene with atomic precision. In this strategy, tailored molecular precursors (often halogenated polycyclic aromatics) are deposited on atomically flat metal surfaces (e.g. Au(111), Ag(111) and Cu(111)) and heated *in vacuo*. Upon mild heating at approximately 150–250°C, the molecules dehalogenate and undergo surface-assisted Ullmann coupling (aryl-aryl C–C bond formation) to form covalent one- or two-dimensional polymeric networks. Subsequent annealing at higher temperatures (approx. 300–400°C) induces cyclodehydrogenation, which planarizes the network into a *sp²*-hybridized graphene sheet or nanoribbon [145,146]. This surface-confined route complements the molecular assembly strategies by offering angstrom-level structural control and a programmable lattice architecture. For example, Pigot & Dumur [147] demonstrated that brominated tetra(biphenyl) monomers on Au(111) polymerize into a crystalline two-dimensional covalent framework (2D-CAP) at approximately 250°C, and further heating yields a planar graphene-like sheet. On-surface synthesis offers unmatched structural control (because precise lattice geometry, edge structure and doping can be programmed using monomers) and can produce atomically narrow nanoribbons or nanographene islands. However, it requires stringent UHV conditions and typically yields only nanoscale domains. By contrast, solution-phase and SAM-based approaches offer complementary advantages that enable the bulk formation of carbon nanosheets (often with a higher defect density), whereas the latter yields atomically precise graphene architectures under ambient-compatible conditions [148]. A complementary solution-processed molecular assembly route is shown in figure 10*I*, where water-dispersible chemically converted graphene (CCG) nanosheets are self-assembled onto a 3-aminopropyl)triethoxysilane (APTES)-functionalized Si wafer through interfacial chemical interactions, forming a monolayer graphene thin film. As illustrated in figure 10*Ia*, surface functionalization promotes uniform nanosheet anchoring, while AFM and TEM images (figure 10*Ib*–*e*) confirm ultrathin, sheet-like morphology consistent with monolayer coverage and good lateral continuity. This study is important in the molecular assembly context because it demonstrates a low-temperature, substrate-directed self-assembly strategy for integrating graphene directly on silicon with strong interfacial binding and good electrical conductivity, highlighting the potential of chemically designed interfaces for scalable graphene/substrate integration [149]. Zhang *et al.* [150] revealed that neutral vinyl monomers can permeate monolayer graphene via the intrinsic defects to trigger surface-initiated controlled radical polymerization (SI-CRP), while the transport of charged monomers is hindered owing to electrostatic interactions with the defect sites. Figure 10*II* shows the transfer of graphene onto a patterned initiator monolayer (panel *IIa*), enabling polymerization beneath graphene after immersion in a monomer solution under a Cu plate (panel *IIb*,*c*). Raman spectra (panel IId) confirm the increased defect density after monomer translocation, and the chemical reaction (panel *IIe*) forms polymer brushes, validating the passage of monomers and growth of polymers across the graphene barrier [150]. Within the UHV-assisted domain, Di Giovannantonio *et al.* [151] elucidated the growth mechanism of 9-atom-wide armchair GNRs (9-AGNRs) via on-surface synthesis on Au(111), emphasizing how halogen functionalization of the monomers (iodine versus bromine) influences the nanoribbon length. As shown in figure 10*IIIa*,*b*, the iodine-containing precursor (DITP) undergoes dehalogenation at lower temperatures, enabling more efficient polymerization and subsequent cyclodehydrogenation (panel *IIIa*), which yields longer GNRs as visualized in the STM image (panel *IIIb*). This two-step strategy highlights how precursor chemistry and thermal activation determine the structural control in bottom-up GNR fabrication [151,155]. Further demonstrating the electronic implications of atomically precise synthesis, Talirz *et al.* reported the bottom-up on-surface synthesis of 9-AGNRs on Au(111) via surface-assisted aryl–aryl coupling and cyclodehydrogenation, yielding atomically precise ribbons with a 1.4 eV bandgap and low carrier effective masses (approx. 0.1 me). The high-resolution non-contact AFM (nc-AFM) image obtained using a CO-functionalized tip (figure 10*IIIc*) clearly shows the atomic lattice of 9-AGNR and confirms its structural precision, which is critical for future nano-electronic applications [152,155]. By contrast, FET studies conducted by Fasel, Bokor and co-workers using 9-AGNR with the lower bandgap revealed significantly improved performance, with a high on-current of 1 µA at a drain voltage of −1 V and a remarkable on-off ratio up to 105 (figure 10*IIId*) [156]. By pushing the boundaries of edge topology control reported by Talirz *et al.* [153], the present authors designed umbrella-shaped monomer **3** with a pre-installed partial zigzag edge, as well as methyl groups that were expected to undergo oxidative cyclization with the neighbouring aromatic rings to form extended zigzag edges (figure 10*IIIe*) [155]. The synthesis of monomer **3** was highly challenging but could be achieved in 14 steps, involving platinum-catalysed cycloaromatization of the ethynyl groups to form a zigzag edge. Monomer **3** allowed the fabrication of the long-awaited zigzag graphene nanoribbons (ZGNRs) with atomically precise structures on an Au(111) surface, as visualized by nc-AFM (figure 10*IIIe*–*g*) [153]. Further characterization of the ZGNRs, and in particular the predicted magnetic properties of the edge states, will be the next challenge, requiring, for example, non-conventional STM with spin-polarized tips or a new method to perform electron spin resonance spectroscopy *in situ* on the Au surface [155]. These molecular assembly strategies offer precise structural control over the graphene architecture, but they are often limited by nanoscale domain sizes, high processing temperatures and the need for carefully tailored molecular precursors.

**Figure 10.**
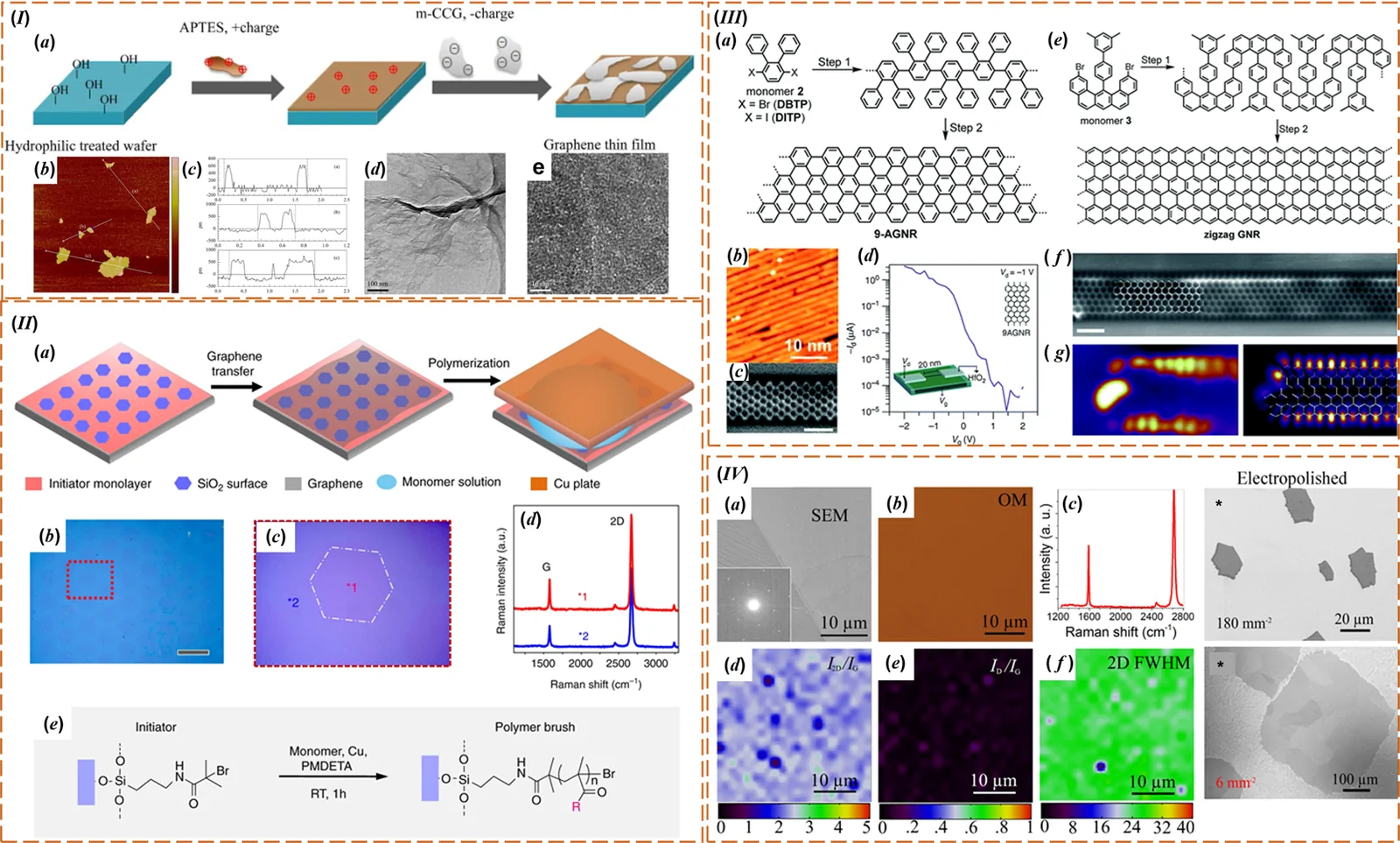
(*I*) (*a*) Schematic for the generation of graphene thin films using self-assembly interaction between graphene nanosheets and the wafer. (*b*) AFM image of CCG after drop-casting on a freshly cleaved mica surface. (*c*) The scanning area for (*a*) is 3 × 3 µm and the *Z* range is 0.8 nm; the height of (*a*–*c*) is 6, 7 and 6 Å, respectively and (*d*,*e*) TEM image of CCG at 100 and 10 nm, respectively [149]. Copyright 2013, Elsevier. (*II*) Substrate preparation and characterization. (*a*) Schematic of the preparation of graphene-BiBB-SiO_2_ and SI-CRP as substrates. (*b*) Optical image of graphene-BiBB-SiO_2_. Scale bar: 40 µm. (*c*) Close-up view of a selected area in (*b*). (*d*) Typical Raman spectra from two positions selected in (*c*). (*e*) Reaction scheme of SI-CRP on graphene-BiBB-SiO_2_. Reproduced with permission [150]. Copyright 2018, Nature Communications. (*III*) On-surface synthesis of GNRs under UHV conditions. (*a*) Synthetic route toward 9-atom-wide armchair GNR (9-AGNR) from monomer **2**. (*b*) STM images showing the length of 9-AGNR obtained from DITP. Reproduced with permission [151]. Copyright 2018, Am. Chem. Soc. (*c*) High-resolution non-contact AFM (nc-AFM) frequency shift image of 9-AGNR using a CO-functionalized tip. Reproduced with permission [152]. Copyright 2017, Am. Chem. Soc. (*d*) *I*_d_–*V*_g_ of the FET devices, showing high *I*_on_ (greater than 1 µA) for a 0.95 nm wide 9-AGNR and high *I*_on_/*I*_off_ (approx. 10^5^). Inset: schematic of scaled 9-AGNR FET device. (*e*) Synthetic route toward a full zigzag GNR (ZGNR) from umbrella-shaped monomer **3**. (*f*) Constant-height nc-AFM frequency shift image of ZGNR taken with a CO-functionalized tip. (*g*) Left: differential conductance maps of filled edge states taken at a sample bias of −0.3 V. Right: DFT-based local density of states at a tip-sample distance of 4 Å, showing the spatial distribution of filled edge states. All scale bars: 1 nm. Reproduced with permission [153]. Copyright 2016, Wiley Adv. Portfolio. (*IV*) (*a*) SEM image of graphene grown on Cu foil. Inset: electron diffraction pattern obtained using TEM. (*b*) Optical microscope image of graphene transferred onto SiO_2_ (300 nm)/Si. (*c*) Typical Raman spectrum of transferred graphene. Raman maps over an area of 40  × 40 µm for (*d*) I_2D_/I_G_ ratio, (*e*) I_D_/I_G_ ratio and (*f*) two-dimensional FWHM. Unit for the colour bar in (*f*): cm^−1^. Two black-and-white images on the rightmost: SEM images after electropolishing and annealed by H_2_ + Ar and in Ar. Reproduced with permission [154]. Copyright 2016, Nature.

### 2.5. Single-atom catalyst method

The synthesis of two-dimensional materials, such as monolayer graphene, demands atomic-level precision because even subtle structural variations can drastically alter the electronic and physical properties [157]. Bottom-up synthetic approaches, particularly those using catalysis, are the most promising methods for achieving precision [158]. SACs, which are typically composed of isolated transition metal atoms, have emerged as the ultimate catalytic system by offering maximal atomic efficiency and potential control over the nucleation and growth of graphene at the atomic scale [159]. *In situ* TEM and STM studies have shown that SACs can add or remove carbon at the edges or defects of graphene with atomic precision. Achieving this level of control requires a fundamental understanding of how individual metal atoms interact with unsaturated carbon bonds at the graphene edges or within vacancy sites. Aberration-corrected TEM plays a pivotal role in directly visualizing such atomic-scale interactions by allowing the identification and tracking of individual atoms within graphene lattices without significant imaging artefacts [160,161].

Several metals, including Cr [32], Ti [162], Fe [162], Pt [162], Ni [163], Al, Au, Ag [164], Co [165] and Si [166], have demonstrated catalytic etching effects on graphene upon electron beam irradiation. For example, Zhao *et al.* [159] used aberration-corrected TEM to observe a single Fe atom hopping along a graphene zigzag edge under an electron beam. The Fe atom was repeatedly bound to a pentagon, captured a C atom from residual hydrocarbons and inserted two C atoms to form hexagons (healing the edge). In other cycles, it ejected carbon to create a vacancy. These pivotal observations highlight the dual catalytic role of single Fe atoms in edge healing and vacancy formation, offering mechanistic insights into atomic-scale control. This study highlights the emerging role of SACs in directing the atomic-scale growth of monolayer graphene. Using both experimental and computational studies, the present authors demonstrated how isolated metal atoms can migrate along the edges of graphene, either in a zigzag or an armchair manner and catalyse the addition of carbon atoms to extend the lattice with atomic precision. Figure 11*a* shows two edge types of graphene, whereas figure 11*b* depicts a single metal atom facilitating edge growth by sequential carbon addition [30]. By contrast, a single Cr atom at the edge only promotes the addition of carbon; new hexagons are formed and the lattice is straightened with no net etching [30]. This study demonstrated that single Cr atoms act as highly efficient catalysts for *sp²* carbon growth at graphene edges, in contrast to most other single atoms that predominantly induce etching. The abovementioned role of the Cr atom is visualized in figure 11*c*–*e*, together with added partial stick-and-ball models to aid viewing (panels *c*′–*e*′), image simulations of the growth process (panels *c*′′–*e*′′), and complete stick-and-ball models (panels *c*′′′–*e*′′′). In figure 11*f*, the blue balls indicate Cr atoms, whereas the red balls and green arrows indicate new C atoms [32].

**Figure 11.**
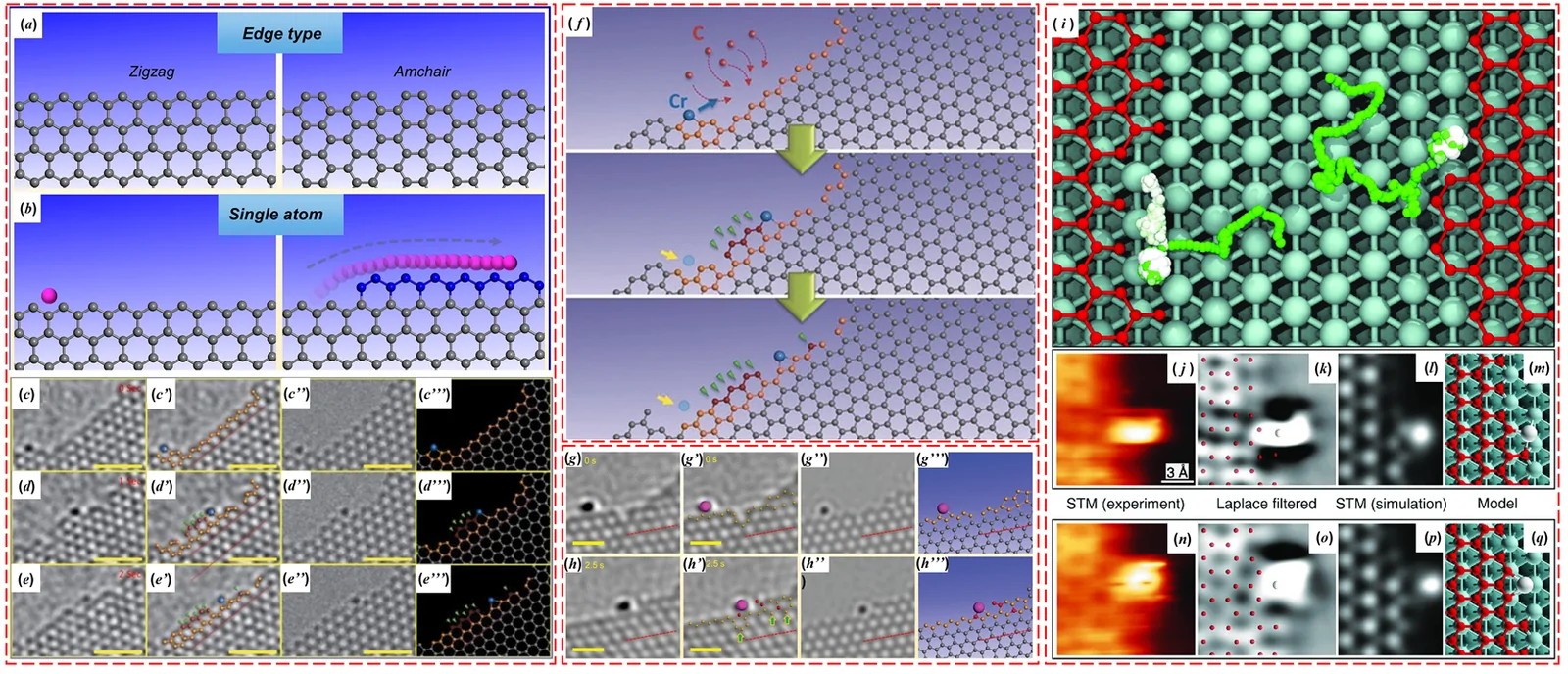
(*a*) Schematic of edge growth from a single catalyst atom on a nanoparticle, showing the (left) zigzag and (right) armchair edge terminations of graphene. (*b*) Schematic of a single-atom edge layer being grown from a SAC. Reproduced with permission [30]. Copyright 2021, Nature npj. (*c*–*e*) Catalytic growth of graphene by a single Cr atom at the graphene edge upon electron beam irradiation. (*c*′,*d*′,*e*′): with added partial stick-and-ball models to aid viewing. (*c*′′,*d*′′,*e*′′): image simulations of the growth process. (*c*′′′,*d*′′′,*e*′′′): complete stick-and-ball models. Blue ball: Cr atom, red balls and green arrows: new C atoms. All scale bars: 1 nm. Nickel adatoms at the graphene edges. Reproduced with permission [32]. Copyright 2018, Springer Nature. (*f*) Schematic of catalytic growth of graphene by a single Cr atom. (*g*,*h*) HRTEM images showing carbon incorporation by a diffusing Sn monomer at a graphene edge upon electron beam irradiation. (*g*′,*h*′): with added ball models to aid viewing. (*g*′′,*h*′′): image simulations of the growth process. (*g*′′′,*h*′′′): complete stick-and-ball models. The red dashed line is included for reference. Dose: approximately 1.8 × 10⁶ A m⁻². All scale bars: 0.5 nm. Reproduced with permission [31]. Copyright 2021, Wiley-AFM. (*i*–*q*) Nickel adatoms at the graphene edges. (*i*) Ni adatoms diffusing on a surface in a region delimited by graphene z (right) and k (left) edges with kinks. Two representative Ni trajectories obtained by molecular dynamics simulations with ReaxFF performed at 710 K for 100 ps are shown. The initial Ni positions are in green and the final positions are in white and also indicated by a larger ball size. (*j*–*q*) Short-lived configurations of Ni adatom at the k-edge kinks. (*j*,*n*) High-speed STM images from movie S2, in the supplementary material; (20) (V = 20 mV, I = 7 nA). (*k*,*o*) Laplace-filtered version of (*j*) and (*n*) with superimposed ball models, respectively. (*l*,*p*) Constant-height STM simulated images based on the calculated geometries (shown in *m* and *q*). Reproduced with permission [167]. Copyright 2018, Science.

A recent study revealed that a single Sn atom can act as a dual-function catalyst, either growing or etching graphene depending on the local carbon feedstock availability. The study also demonstrated the dynamic behaviour of Sn dimers at graphene edges. Panels (*g*, *h*, *g*′, *h*′) in figure 11 show HRTEM snapshots with overlaid models tracking Sn-catalysed C incorporation into the graphene lattice, while panels (*g*′′, *h*′′, *g*′′′, *h*′′′) show simulated and modelled atomic configurations that highlight how individual C atoms are added to the edge under electron beam stimulation [31]. This dual behaviour contrasts with the exclusive growth role of Cr and underscores the influence of feedstock environment on SAC functionality. A complementary investigation by Patera *et al.* [167] using STM and DFT simulations demonstrated that single Ni adatoms catalyse graphene growth at kinked edges on Ni(111) by inserting carbon atoms into incomplete rows, as revealed by millisecond-resolution STM. Figure 11*i* shows the diffusion trajectories of the Ni adatom at the graphene edges, and figure 11*j*–*q* shows a sequence of experimental STM snapshots, image filtering, simulated STM and structural models, collectively capturing the transient configurations of Ni at kink sites during active graphene edge propagation [167]. Similarly, the STM/DFT study in [30] showed that Ni adatoms dwell at kink-edge sites and catalyse orderly row-by-row C insertion during growth. Conversely, aberration-corrected STEM imaging of suspended graphene with evaporated metals revealed that isolated Pd, Ti and Al atoms (and Ni or Cr adatoms under some conditions) readily initiate hole formation. Specifically, each metal (except Au) drastically lowers the formation energy of a carbon vacancy (especially in the presence of trace O₂/H₂O), effectively ‘drilling’ nanoscale pores under the beam [168]. Such destructive dynamics highlight the dual potential of SACs for the construction and/or degradation of graphene under external conditions [159,166]. These findings underscore the transformative potential of SACs in precision graphene fabrication, where each catalytic event is governed by a single atom [30].

Taken together, the routes surveyed above occupy distinct positions in the trade-off between crystalline quality, monolayer selectivity and scalability, and these differences are reflected in their characteristic quality metrics. Table 2 compiles representative quantitative indicators for each approach, including the Raman defect ratio (I_D_/I_G_), the monolayer fingerprint (I_2D_/I_G_), lateral domain size and coverage, carrier mobility and specific surface area (SSA). Metal-catalysed and SiC-epitaxial CVD deliver the lowest defect densities (I_D_/I_G_ well below 0.1) together with centimetre- to millimetre-scale single-crystal monolayers, whereas arc discharge yields comparatively defective, predominantly few-layer flakes (I_D_/I_G_ ≈ 0.6–0.9) in which true monolayers are rare. Molecular and on-surface assembly trades macroscopic continuity for atomic precision, producing nanocrystalline films or atomically defined nanoribbons rather than wafer-scale monolayers, while SAC-mediated growth currently exists only as single-atom, electron-beam-driven events that are not yet captured by ensemble metrics. It is worth noting that the relevance of each descriptor is route-dependent: SSA, which approaches a theoretical maximum of approximately 2630 m² g⁻¹ for an ideal single layer, is a meaningful quality benchmark for flake- and powder-type products but is not the operative figure of merit for continuous films, where domain size, (I_D_/I_G_), and carrier mobility better capture device-grade quality. This comparative perspective underscores that no single route simultaneously optimizes all metrics, motivating the hybrid and AI-assisted strategies discussed below.

**Table 2.** Representative quantitative quality metrics for the principal bottom-up routes to monolayer graphene. Values are best-case or typical figures reported in the cited studies; ‘N/A’ denotes a metric not conventionally defined for that route. The Raman defect ratio I_D_/I_G_ and the monolayer signature I_2D_/I_G_ are measured by Raman spectroscopy. The theoretical specific surface area (SSA) of defect-free SLG is approximately 2630 m² g⁻¹, against which flake/powder products (e.g. arc discharge) are benchmarked; for continuous films (CVD, SiC epitaxy and on-surface assembly), domain size, I_D_/I_G_ and carrier mobility are the operative quality descriptors.

synthesis route			lateral domain size & monolayer coverage	SSA	ref.
CVD metal-catalysed (Cu, Cu(111), Cu–Ni, Ni–Mo, Ni(111))	I_D_/I_G_ ≈ 0.076 (near-defect-free, graphene/Pd leaf), generally ≤ 0.1 for device-grade monolayer	I_2D_/I_G_ > 2 (often ≈ 3); sharp single-Lorentzian 2D band ⇒ monolayer	cm-scale single crystal on Cu(111)/sapphire, single-crystal Cu(111) tape > 1 m (roll-to-roll) > 90% monolayer coverage on Cu(111), Cu/Ni(111) full monolayer in 5 min, Ni–Mo 100% coverage, Ni(111) 91.4% vs poly-Ni 72.8%	continuous film—SSA not the standard QC metric	[44–46,60,61,64,65,77]
CVD — transfer-free / direct on dielectrics & insulators (SiO₂, sapphire, Si₃N₄, h-BN)	low, but usually higher than on metal, minimal D under O₂-/H₂O-/CO₂-assisted growth	monolayer 2D signature reported, I_2D_/I_G_ mapping used to confirm uniformity	wafer-scale (4-inch) via patterned Pt seed; SLG on H₂-etched sapphire (≈ 73 meV gap at Dirac point); polycrystalline on Si₃N₄	films, SSA N/A	[76,78,86,103,104,106]
epitaxial growth on SiC (TSA, IECS, PASG, 3C-SiC/Si)	low (minimal D-band): monolayer–bilayer with negligible D (TSA), minimal D (IECS)^a^.	high, clear monolayer/bilayer 2D; high I_2D_/I_G_ under optimized IECS.	mm-scale single-crystal monolayer (2 × 2 mm on 4H-SiC Si-face), wafer-scale; monolayer approximately 1200°C, up to 8 layers at higher T on 3C-SiC/Si	film on wafer, SSA N/A	[123,125,128,129,169]
arc / plasma discharge	comparatively high defective, multi-layer, typically ≈ 0.6–0.9, chamber-/gas-dependent; He plasma → fewer defects than Ar.	low I_2D_/I_G_ (approx. 0.5–0.6) ⇒ few-/multi-layer; true monolayer signature rare	flakes 3–10 layers, lateral approximately 1–10 µm; monolayers rare (< 1% of flakes), He favours monolayer-rich but low yield	flake/powder product, SSA measurable and meaningful, route-dependent.	[21,134,136,137,170]
molecular / on-surface assembly (SAM pyrolysis, liquid-interface, UHV GNR synthesis)	nanocrystalline films: pronounced D band, I_D_/I_G_ approximately 1 (no long-range order); on-surface GNRs show intrinsic edge-derived D band (not a disorder metric).	weak/broadened 2D for nanocrystalline films; GNRs show characteristic width-dependent modes.	µm-scale sheets at liquid interface; suspended monolayer membranes approximately 250 µm²; GQDs approximately 1.3 nm; GNRs atomically precise, nm-wide, lengths to approximately µm	high for nanographene/porous networks (approaches SLG values for ideal monolayers), not standardized.	[134,140,142,143,152, 153,155,156]
SAC-mediated growth/ etching	not established, characterized by aberration-corrected TEM/STM at the single-atom level, not by ensemble Raman, I_D_/I_G_ undefined for single-event demonstrations.	N/A (no macroscopic film)	single-atom / single-edge events, no continuous film yet	N/A (single-event, sub-nm observations)	[30–32,159,167,168]

### 2.6. Artificial intelligence and machine learning-assisted strategies

To complement the experimental advances, researchers increasingly turn to AI and ML tools to analyse, predict and control graphene synthesis [171]. ML is being integrated into the synthesis and post-processing of graphene to accelerate material discovery, optimize growth conditions and automate quality control. ML models can learn complex, nonlinear relationships between the CVD parameters and the resulting film characteristics, enabling accurate predictions of grain size, coverage and defect density [172]. Convolutional neural networks automatically quantify domain morphology from the SEM/optical images, whereas generative adversarial networks and other deep-learning frameworks can visualize how changes in process variables alter the grain structure.

On the CVD front, Hwang *et al.* [173] developed a data-driven pipeline, in which a convolutional neural network automatically quantifies grain morphology from SEM images, followed by artificial neural networks and support vector machines that map process parameters to key film metrics (i.e. size, coverage, domain density and aspect ratio). A generative adversarial network then translates these predictions into realistic graphene microstructures, allowing rapid ‘virtual experiments’ to identify growth conditions that yield large domains and low defect density. Complementing this predictive framework, Yoshihara *et al.* [172] trained Gaussian-process regression models to link the surface spectra of Cu foil and CVD conditions to graphene domain size. Using process parameters suggested by the trained model, those authors produced single-crystal domains with a size of approximately 1.3 mm, far larger and faster compared with conventional recipes. Support vector machines, Gaussian processes and other regression models suggest optimal growth conditions, reduce the amount of trial-and-error, and accelerate the synthesis of millimetre-scale single-crystal domains.

Beyond growth, AI algorithms also aid post-synthesis inspection. Defect detection networks classify wrinkles, cracks and residues on transferred graphene and provide rapid quality control [174]. Also, Sabet combined CVD with microwave reduction and used neural networks to optimize hybrid-graphene electrodes for supercapacitors, achieving high conductivity (approx. 9.6 × 10⁵ S m⁻¹), large specific cap acitance (500 F g⁻¹), and long-term stability [175]. As researchers integrate ML with real-time sensor data and closed-loop control, AI-guided process design promises to deliver scalable, high-quality monolayer graphene, complementing advances in the synthetic approaches (CVD, epitaxial growth, arc discharge, molecular assembly and single-atom catalysis). Collectively, these studies demonstrate that coupling AI/ML with experimental and computational techniques can accelerate parameter exploration, identify optimal growth windows, automate defect detection and ultimately lower the cost and time required to produce high-quality monolayer graphene.

## 3. Future outlook

Although bottom-up methods have brought monolayer graphene close to practical deployment, formidable challenges remain in controlling domain size, lattice perfection and cost at the wafer scale. For CVD, the next breakthroughs will probably come from self-limiting growth on non-metal catalysts and real-time closed-loop process control. Developing catalytic coatings that can be annealed away would enable transfer-free monolayers. *In situ* spectroscopic monitoring combined with ML algorithms can dynamically adjust the temperature, flow and precursor chemistry to minimize thickness variations and wrinkles. For SiC epitaxy, polymer-assisted sublimation and thin 3C-SiC/Si templates show promise for lowering sublimation temperatures and costs. Coupling these approaches with surface-energy engineering (e.g. step-edge templating and doped buffer layers) may suppress multi-layer formation while harnessing the unique electronic properties of EG. Arc-discharge synthesis remains underexplored relative to CVD and SiC, and future work should focus on: (i) stabilizing the plasma to increase monolayer yield, (ii) optimizing ambient gases (He/H₂ mixtures) to favour few-layer growth, and (iii) designing hybrid arc-CVD reactors that combine plasma-generated radicals with catalytic surfaces. Molecular assembly holds the key to atomic precision. Research efforts should aim to bridge the gap between nanometre-scale nanoribbons and macroscopic sheets by designing precursors that self-align on catalytic templates and developing scalable cross-linking/pyrolysis protocols. SACs have demonstrated atomic-level growth and etching in TEM. Translating these single-event observations into practical synthesis will require a deeper understanding of adatom migration, edge kinetics and feedstock supply, possibly through multi-scale modelling and tailored supports.

In all synthetic methods, AI/ML will play a central role in accelerating discovery and process optimization. Neural networks and Gaussian process models can map nonlinear relationships between process parameters and film properties. Generative models can virtually screen morphologies and suggest growth windows, and defect recognition algorithms can automate quality control. For example, reinforcement learning approaches may eventually guide atomic-scale growth decisions in SAC systems or help reduce transfer-induced defects in CVD processes. As more experimental data become available, AI-driven models will significantly accelerate the path toward reproducible wafer-scale monolayer graphene with fewer trial-and-error iterations. Ultimately, the way for industrially viable, defect-free monolayer graphene will be facilitated by (i) integrating *in situ* diagnostics with AI-driven feedback, (ii) leveraging low-temperature and environmentally friendly chemistries, and (iii) combining the scalability of CVD, direct integration of SiC epitaxy, purity of arc discharge, precision of molecular assembly and atomic control of SACs.

## 4. Conclusions

The synthesis of monolayer graphene has progressed from empirical exfoliation to a suite of bottom-up strategies that balance scalability against atomic precision. CVD remains the most scalable route, delivering centimetre-scale single-crystal domains on Cu via innovative approaches, such as single-crystal foils, roll-to-roll annealing and dry transfer. However, post-growth transfer still introduces cracks, wrinkles and contamination. Epitaxial growth on SiC offers transfer-free integration and wafer-scale uniformity through controlled silicon sublimation, hydrogen etching, argon confinement and TSA. Yet, its adoption is constrained by high temperatures (greater than 1500°C), expensive wafers and persistent multi-layer regions, prompting research into polymer-assisted sublimation and thin 3C-SiC/Si templates. Arc discharge methods enable simple, catalyst-free production of crystalline graphene flakes with minimal metal contamination. However, the monolayer yield is low and highly sensitive to the discharge current, gas composition, chamber geometry and coproduction of amorphous carbon/nanotubes. These issues have limited the product purity, although hybrid arc/CVD and aqueous arc methods hint at improved control. Molecular assembly through the pyrolysis of cross-linked SAMs or cyclodehydrogenation of designer precursors has enabled the development of nanoribbons and porous sheets with unrivalled atomic precision, offering tuneable widths, edge chiralities and controlled doping. Nevertheless, scaling these atomically precise architectures beyond micrometre dimensions and avoiding UHV conditions remain major challenges. The SACs epitomize atomic-scale control. Aberration-corrected TEM and STM studies have shown that isolated Fe, Cr, Ni, Sn and other adatoms guide carbon addition or removal of one atom at a time, enabling edge repair, growth and etching with unprecedented precision. Specifically, Cr exclusively catalyses growth, Sn and Fe mediate both growth and etching, whereas Pd, Ti and Al favour vacancy formation. Although the SACs currently operate only as single events under electron beams, they signal a transformative direction toward atomically engineered graphene.

On the computational front, ML and AI tools are essential for solving the problems encountered in various synthetic routes. Data-driven models are already being used to map the complex relationships between CVD parameters and film properties, predict optimal growth conditions and visualize expected morphologies without exhaustive experimentation. AI-enabled defect-detection systems automate the inspection of transfer-induced flaws, whereas the ML-guided optimization of hybrid synthesis routes (such as CVD combined with microwave reduction) delivers high-performance graphene electrodes for energy storage. These tools accelerate discovery, reduce trial-and-error cycles and support real-time closed-loop process controls. The future of monolayer graphene synthesis will integrate the scalability of CVD, direct integration of SiC epitaxy, catalyst-free purity of arc discharge, structural programmability of molecular assembly, atomic precision of SACs and predictive power of AI/ML supported by *in situ* diagnostics, computational modelling, greener feedstocks and lower-temperature processes to realize industrially viable, defect-free graphene with self-controllable properties.

## Data Availability

This article has no additional data.
